# Augmented ERO1α upon mTORC1 activation induces ferroptosis resistance and tumor progression via upregulation of SLC7A11

**DOI:** 10.1186/s13046-024-03039-2

**Published:** 2024-04-13

**Authors:** Zixi Wang, Huaiyuan Zong, Weiwei Liu, Wei Lin, Anjiang Sun, Zhao Ding, Xu Chen, Xiaofeng Wan, Yanyan Liu, Zhongdong Hu, Hongbing Zhang, Hongwu Li, Yehai Liu, Dapeng Li, Sumei Zhang, Xiaojun Zha

**Affiliations:** 1 Department of Biochemistry & Molecular Biology, School of Basic Medicine, Anhui Medical University, No. 81 Meishan Road, Hefei, 230032 Anhui Province China; 2 Department of Otorhinolaryngology, Head & Neck Surgery, The First Affiliated Hospital of Anhui Medical University, Hefei, 230022 China; 3 Department of Stomatology, The First Affiliated Hospital of Anhui Medical University, Hefei, 230022 China; 4 Hefei Cancer Hospital, Chinese Academy of Sciences, Hefei, 230031 China; 5 Department of Thyroid and Breast Surgery, Hefei First People’s Hospital, Hefei, 230061 China; 6 Modern Research Center for Traditional Chinese Medicine, Beijing Research Institute of Chinese Medicine, Beijing University of Chinese Medicine, Beijing, 100029 China; 7 State Key Laboratory of Medical Molecular Biology, Department of Physiology, Institute of Basic Medical Sciences and School of Basic Medicine, Peking Union Medical College and Chinese Academy of Medical Sciences, Beijing, 100730 China; 8 Anhui Public Health Clinical Center, Hefei, 230011 China; 9 Department of Otorhinolaryngology, Head & Neck Surgery, The Affiliated Bozhou Hospital of Anhui Medical University, No. 616 Duzhong Road, Bozhou, 236800 Anhui Province China; 10 Children’s Hospital of Fudan University, National Children’s Medical Center, And Institutes of Biomedical Sciences, Fudan University, Shanghai, 200032 China

**Keywords:** mTOR, Ferroptosis, ERO1α, SLC7A11, Tumor growth

## Abstract

**Background:**

The dysregulated mechanistic target of rapamycin complex 1 (mTORC1) signaling plays a critical role in ferroptosis resistance and tumorigenesis. However, the precise underlying mechanisms still need to be fully understood.

**Methods:**

Endoplasmic reticulum oxidoreductase 1 alpha (ERO1α) expression in mTORC1-activated mouse embryonic fibroblasts, cancer cells, and laryngeal squamous cell carcinoma (LSCC) clinical samples was examined by quantitative real-time PCR (qRT–PCR), western blotting, immunofluorescence (IF), and immunohistochemistry. Extensive in vitro and in vivo experiments were carried out to determine the role of ERO1α and its downstream target, member 11 of the solute carrier family 7 (SLC7A11), in mTORC1-mediated cell proliferation, angiogenesis, ferroptosis resistance, and tumor growth. The regulatory mechanism of ERO1α on SLC7A11 was investigated via RNA-sequencing, a cytokine array, an enzyme-linked immunosorbent assay, qRT–PCR, western blotting, IF, a luciferase reporter assay, and a chromatin immunoprecipitation assay. The combined therapeutic effect of ERO1α inhibition and the ferroptosis inducer imidazole ketone erastin (IKE) on mTORC1-activated cells was evaluated using cell line-derived xenografts, LSCC organoids, and LSCC patient-derived xenograft models.

**Results:**

ERO1α is a functional downstream target of mTORC1. Elevated ERO1α induced ferroptosis resistance and exerted pro-oncogenic roles in mTORC1-activated cells via upregulation of SLC7A11. Mechanically, ERO1α stimulated the transcription of SLC7A11 by activating the interleukin-6 (IL-6)/signal transducer and activator of transcription 3 (STAT3) pathway. Moreover, ERO1α inhibition combined with treatment using the ferroptosis inducer IKE exhibited synergistic antitumor effects on mTORC1-activated tumors.

**Conclusions:**

The ERO1α/IL-6/STAT3/SLC7A11 pathway is crucial for mTORC1-mediated ferroptosis resistance and tumor growth, and combining ERO1α inhibition with ferroptosis inducers is a novel and effective treatment for mTORC1-related tumors.

**Supplementary Information:**

The online version contains supplementary material available at 10.1186/s13046-024-03039-2.

## Introduction

The serine/threonine protein kinase mechanistic target of rapamycin (mTOR) is essential for regulating the fundamental biological processes of cell growth and survival [1]. mTOR signaling is mediated by two branches, mTOR complex 1 (mTORC1) and mTOR complex 2 (mTORC2), which are different in terms of specific binding partners, upstream and downstream signaling, and the response to rapamycin [2]. mTORC1 regulates several biological processes important for cell growth and metabolism by integrating numerous signals such as growth factors, amino acids, and energy sources [3, 4]. The tuberous sclerosis 1 (TSC1)/tuberous sclerosis 2 (TSC2) complex is a well-known upstream suppressor of mTORC1 signaling [5]. Depending on the GTPase activity of TSC2, the TSC1/TSC2 complex inhibits the Ras homolog enriched in the brain (Rheb), a small GTPase, by promoting the conversion of active Rheb-GTP to inactive Rheb-GDP [6]. Dissociation of the TSC1/TSC2 complex by inactivating mutations in either *TSC1* or *TSC2* genes or by the phosphorylation of TSC2 with protein kinase B (AKT) results in the accumulation of Rheb-GTP, which ultimately switches on mTORC1 signaling [7]. mTORC1 enhances protein synthesis primarily via the phosphorylation of two key downstream effectors, ribosomal protein S6 kinase 1 (S6K1) and eukaryotic translation initiation factor 4E-binding protein 1 (4E-BP1), and then promotes cell growth and proliferation [1]. Accumulating evidence indicates that mTORC1 signaling is frequently over-activated in human cancers [8, 9], but the precise mechanisms still require further elucidation.

Ferroptosis is a recently identified type of cell death driven by iron-dependent lipid peroxidation [10]. In terms of morphology, its typical manifestations include increased mitochondrial membrane density, a significant reduction of cristae, and mitochondrial shrinkage [10]. Lipid peroxidation is the core reaction of ferroptosis, which is a process that leads to oxidative degradation of lipids, resulting in the formation of peroxide and hydroperoxide derivatives [11]. The main primary products of lipid peroxidation are lipid hydroperoxides. Among the secondary products of lipid peroxidation, malondialdehyde (MDA) is believed to be the most mutagenic product, whereas 4-hydroxynonenal (4-HNE) is the most toxic [12]. Several ferroptosis defense systems have been found in cells to maintain cell survival by counteracting the adverse effects of lipid peroxidation [10, 13]. The glutathione peroxidase 4 (GPX4)/Glutathione (GSH) system is the primary cellular defense against ferroptosis [14]. Member 11 of the solute carrier family 7 (SLC7A11), an upstream molecule of the GPX4/GSH system, plays a vital role in resisting ferroptosis [15]. It mediates the intake of cystine, which is then reduced to cysteine within the cell and participates in GSH synthesis [15]. GPX4 specifically catalyzes the loss of oxidative activity of lipid peroxides in a GSH-dependent manner, thereby inhibiting ferroptosis [16]. Compounds such as erastin (an inhibitor of SLC7A11) or RAS-selective lethal 3 (RSL3, a GPX4 inhibitor) can induce ferroptosis, while antioxidants, such as ferrostatin-1 and liproxstatin-1 (Lip-1), can suppress ferroptosis [17]. A growing amount of preclinical evidence suggests that inducing ferroptosis is an effective way to treat tumors [18, 19].

Recently, mTORC1 was found to act as a negative regulator of ferroptosis in cancer cells [20]. For example, Zhang et al. reported that cyst(e)ine promotes GPX4 protein synthesis by activating mTORC1 signaling, thus enhancing ferroptosis resistance to RSL3 [21]. It has also been reported that mTORC1 promotes de novo monounsaturated fatty acid synthesis and ferroptosis resistance by upregulating the sterol regulatory element-binding protein 1/stearoyl-CoA desaturase 1 axis [22]. Nevertheless, the exact mechanisms by which mTORC1 promotes ferroptosis resistance remain largely uncharacterized.

In this study, by analyzing mTORC1-activated mouse embryonic fibroblasts (MEFs) and cancer cells, laryngeal squamous cell carcinoma (LSCC) clinical samples, LSCC patient–derived organoids (PDO), and LSCC patient–derived xenograft (PDX) models, we established that endoplasmic reticulum oxidoreductase 1 alpha (ERO1α) is involved in hyperactivated mTORC1-mediated ferroptosis resistance and tumor growth. We also demonstrated that ERO1α upregulates SLC7A11 by activating the interleukin-6 (IL-6)/signal transducer and activator of transcription 3 (STAT3) pathway, thus promoting ferroptosis resistance and tumoral growth of mTORC1-activated cells. Moreover, we showed that ERO1α inhibition sensitizes mTORC1-activated cells or tumors to the ferroptosis inducer imidazole ketone erastin (IKE)*.* We posit that the ERO1α/IL-6/STAT3/SLC7A11 pathway is critical for mTORC1-mediated ferroptosis resistance and tumor growth and that it can be targeted for the treatment of cancers associated with dysregulated mTORC1 signaling.

## Materials and methods

### Reagents and antibodies

Rapamycin (a mTORC1 specific inhibitor), MHY1485 (a mTOR activator), S3I-201 (a selective STAT3 inhibitor), IKE, and Lip-1 were purchased from Selleck Chemicals (TX, USA). Puromycin and deferoxamine (DFX) were sourced from Sigma-Aldrich (MO, USA). Erastin, recombinant mouse IL-6, monokine induced by interferon-gamma (MIG), and macrophage inflammatory protein-1 alpha (MIP-1α) proteins were obtained from Med Chem Express (NJ, USA). Plasmids such as pBabe-puro, pBabe-puro-STAT3C (constitutively activated STAT3), pGL3-Basic, and pRL-TK were previously reported [23]. Lenti-CRISPRv2, pMD2.G, and psPAX2 plasmids were obtained from Addgene (MA, USA). The antibodies used in this article were listed in Supplementary Table S1.

### Cell lines and cell culture

All MEF cell lines (Tsc1 + / + , Tsc1 − / − , Tsc2 + / + , and Tsc2 − / −) have been described previously [23, 24]. The human umbilical vein endothelial cells (HUVECs), human embryonic kidney 293 T (HEK 293 T) cells, human pancreatic carcinoma cells (PANC-1), human breast carcinoma cells (MDA-MB-231), human lung adenocarcinoma cells (A549), and human cervical cancer cells (HeLa) were obtained from the American Type Culture Collection (VA, USA). LSCC cell lines (Raptor-knockdown LIU-LSC-1 cells, TSC2-knockout TU177 cells, and their respective controls) were described in a previous study [25]. MEFs, HUVECs, HEK 293 T, PANC-1, MDA-MB-231, A549, and HeLa cells were cultured in DMEM Medium (BOSTER, Wuhan, China) supplemented with 10% fetal bovine serum (Gibco, CA, USA) and 1% penicillin–streptomycin (Beyotime, Jiangsu, China). LSCC cell lines were cultured in RPMI 1640 medium (BOSTER) with the same composition as MEFs. Cell cultures were frequently monitored for mycoplasma contamination, and only mycoplasma-negative cells were used for experiments.

### Clinical specimens

LSCC tissues and adjacent normal mucosal (ANM) tissues were collected from the First Affiliated Hospital of Anhui Medical University during 2021 to 2022 with patient consent. Prior to surgery, all recruited patients had not undergone chemotherapy, radiotherapy, or any other antitumor therapies. Histological diagnosis of LSCC was confirmed by at least two experienced pathologists for all samples. The use of tissue samples in this study was approved by the Research Ethics Committee of the First Affiliated Hospital of Anhui Medical University. Detailed patient information was provided in Supplementary Table S2.

### Establishment of NTC/T1 null cells

A subcutaneous tumor, formed by the injection of Tsc1 − / − MEFs into BALB/c-nude mice (GemPharmatech, Nanjing, China), was isolated and immediately washed with cold phosphate-buffered saline (PBS) for 3 times. Subsequently, the tumor tissue was cut into small pieces, and then the small tumor masses were enzymatically dissociated with DMEM medium containing 200 U/ml type IV collagenase (Sigma-Aldrich) at 37 °C for 12 h. Afterward, the cells were washed with PBS and centrifuged, and the sediments were seeded into 6 cm petri dishes and cultured in DMEM medium containing 10% fetal bovine serum (Gibco). Cells were passaged every 3–4 days to prevent cell confluency. Cancer-associated fibroblasts were removed by brief exposure to a 0.25% trypsin solution (Beyotime). This newly established cell line was named NTC/T1 null, and the deletion status of TSC1 in the cells was confirmed by western blotting.

### Generation of a ERO1α knockout (KO) cell line using CRISPR-Cas9

Ero1α KO NTC/T1 null cell lines were generated following a protocol from the Feng Zhang lab (https://zhanglab.bio) using Lenti-CRISPRv2 plasmids. The sgRNA target sequence for Ero1α was 5'-GTAGTTATTAAACTTATCGA-3'. Lentivirus was produced by co-transfecting the recombinant viral plasmids with packaging vectors (pMD2.G and psPAX2) into HEK 293 T cells. The virus-containing supernatant was harvested 48 h after transfection and infected with NTC/T1 null cells, followed by selection with puromycin for 1 week. Then, the cells were trypsinized, diluted to single cells, and seeded into 96-well plates. Positive single clones were identified by western blotting. An empty Lenti-CRISPRv2 vector was used to generate a control cell line.

### Lentivirus production and transduction

All of the lentiviral vectors were provided by GenePharma (Shanghai, China), which included the LV4 lentiviral plasmid expressing ERO1α cDNA, SLC7A11 cDNA, and the empty plasmid; LV-2N lentiviral shRNA expression vector targeting ERO1α, SLC7A11, and the control scrambled shRNẠ (shSc). The detailed information of the target sequences was listed in Supplementary Table S3. Lentivirus production and the generation of stable cell lines have been described previously [23].

### Western blotting

Western blotting was performed as described previously [26]. In brief, cell or tissue lysates were separated using NuPAGE 4–12% Bis–Tris gels (Life Technologies, CA, USA) and subsequently transferred onto PVDF membranes (Millipore, MA, USA). Following blocking with 5% skim milk, the membranes were incubated with primary and secondary antibodies. Finally, the protein bands were visualized using Pierce™ ECL Western Blotting Substrate (Thermo Fisher Scientific, MA, USA) with a ChemiScope 6100 instrument (Clinx, Shanghai, China).

### Quantitative real-time PCR (qRT–PCR)

Total RNA was extracted from cultured cells using TRIzol reagent (Invitrogen, CA, USA) following the manufacturer’s instructions. The quality and concentration of the extracted RNA were assessed using NanoDrop 2000 (Thermo Fisher Scientific). 1 μg of total RNA was transcribed into first-strand cDNA using a RevertAid™ First Stand cDNA Synthesis Kit (Fermentas, MA, USA). qRT–PCR was performed using SYBR Premix Ex TaqTM II (TaKaRa, Kyoto, Japan) in qPCR system LightCycler® 96 (Roche, Switzerland) according to the manufacturer’s protocol. Expression levels of the target genes were normalized to β-actin using the 2^−ΔΔCt^ formula. The primer sequences were provided in Supplementary Table S4.

### RNA interference

All siRNA oligonucleotides were synthesized by GenePharma. Cells were transfected with siRNA at a confluence of 50–60% using GP-transfect-mate (GenePharma) according to the manufacturer’s protocol. The target sequences were provided in Supplementary Table S5.

### Tsc2 + / − mice

Tsc2 + / − mice were described previously and backcrossed to Balb/c strain for over 10 times [27]. Toes were used for DNA extraction and genotyping. Kidneys were collected from ten-month-old mice upon sacrifice for subsequent immunohistochemistry analysis.

### Reporter constructs and luciferase reporter assay

The genomic region containing the mouse *SLC7A11* promoter (− 575 to + 144) was amplified by PCR and then cloned into the pGL3-Basic vector to construct the *SLC7A11* promoter luciferase reporter. Potential STAT3 binding sites within the promoter of the mouse *SLC7A11* gene were mutated using the Q5 Site-Directed Mutagenesis Kit (NEB, MA, USA). Primers sequences were listed in Supplementary Table S6.

To determine the impact of STAT3 on the transcription of *SLC7A11*, HEK 293T cells were seeded in 24-well plates and co-transfected with wild-type or mutated promoter constructs (200 ng), along with pBabe-puro-STAT3C (200 ng) or the empty vector pBabe-puro and the internal control pRL-TK (10 ng). Luciferase activity was analyzed using the Dual-Luciferase Reporter Assay System (Promega, WI, USA) following the manufacturer's protocol. Relative luciferase activity was normalized to the Renilla luciferase internal control.

### Immunofluorescence (IF) staining

Cells (2 × 10^4^) were seeded in 35 mm glass bottom culture dishes (Thermo Fisher Scientific) and incubated overnight. After fixed with 4% paraformaldehyde, permeabilized with 0.1% Triton X-100, and blocking with 3% bovine serum albumin, the cells were stained with primary antibodies overnight at 4 °C, followed by incubation with Alexa Fluor 647-conjugated or CY3-conjugated secondary antibodies (Beyotime) for 1 h. The dishes were counterstained for cell nuclei with DAPI (Beyotime). Fluorescent images were acquired using an LSM880 + Airyscan confocal laser scanning microscope (Carl Zeiss, Oberkochen, Germany).

### Transmission electron microscopy (TEM) assay

Cells were seeded in 10 cm dishes and treated with erastin for 16 h. After washing with cold PBS, the cells were fixed with 2.5% glutaraldehyde in phosphoric acid buffer for 2 h, followed by additional fixation in 1% osmium tetroxide for 2 h. And then, cell sections were dehydrated in ethanol and embedded in acetone. The sections, with a thickness of 70 nm, were stained with uranium acetate and lead citrate. TEM images were captured using the transmission electron microscope (JEOL, Tokyo, Japan).

### MDA assay

The level of MDA was determined using a Lipid Peroxidation MDA Assay Kit (Beyotime). In brief, cells were plated at approximately 80% confluency into 6 cm dishes. After pre-treatment with erastin for 24 h, the cells were lysed, and the supernatant was collected following centrifugation. Tumor tissues were homogenized and sonicated in RIPA buffer (Beyotime) on ice, and then the supernatant was collected by centrifuging at 12,000 × g for 15 min at 4 °C. Protein concentrations of the cell and tissue lysates were measured using a BCA Protein Assay Kit (Beyotime). MDA levels in the collected cell and tissue supernatant were measured following the manufacturer’s instructions.

### L-ROS assay

Cells were seeded at a density of 60–70% confluence into 6-well plates. After treatment with erastin for 24 h, the cells were stained with 2 μM C11 BODIPY (581/591) (Thermo Fisher Scientific) for 25 min at 37 °C. Subsequently, the cells were washed, trypsinized, and resuspended in 300 µl fresh PBS. Next, the cells were passed through a 40-µm cell strainer and analyzed with a flow cytometer (Beckman Coulter, CA, USA). Oxidation of BODIPY C11 resulted in a shift of the fluorescence emission peak from 590 to 510 nm. Data was analyzed using Cytobank (https://www.cytobank.cn/).

### GSH assay

A total of 1 × 10^6^ cells were seeded into 6 cm culture dishes. After treatment with erastin for 24 h, the cells were harvested for the quantification of GSH and GSSG using a GSH and GSSG Assay Kit (Beyotime) according to the manufacturer's protocol. The total GSH and GSSG levels were calculated using a standard curve and normalized to the total protein content, determined using the BCA method. The following formula: reduced GSH = total glutathione (GSH + GSSG) – GSSG × 2, was used to calculate the concentration of reduced GSH.

### Chromatin immunoprecipitation (ChIP)

A ChIP assay was conducted using a SimpleChIP® Plus Enzymatic Chromatin IP kit (Cell Signaling Technology, MA, USA) according to the protocol provided by the manufacturer. In brief, cells were crosslinked with 1% formaldehyde, and chromatin was extracted and sonicated to an average size of 200–500 bp using an Ultrasonic crusher (Thermo Fisher Scientific). Subsequently, the samples were incubated with an anti-STAT3 Tyr^705^ antibody (Cell Signaling Technology) overnight at 4 ℃. The immunoprecipitated DNA was purified and analyzed by PCR or qRT–PCR using specific primers. The primer sequences were listed in Supplementary Table S7.

### Cell viability, EdU staining and Colony formation assays

Cell proliferation was evaluated using the Cell Counting Kit-8 (CCK-8) kit (Beyotime). Cells were seeded onto 96-well plates at 2 × 10^3^ cells/well density. The 10 μL of CCK-8 solution was added to each well at the indicated time points following the manufacturer’s instructions, and incubated for 2 h. The OD value at 450 nm was detected for each well using a microplate reader (Thermo Fisher Scientific).

Cell viability was also determined using the CCK-8 kit. Cells were seeded at a density of 1 × 10^4^ cells per well in 96-well plates. After incubating 12 h, the cells were treated with erastin at the indicated concentrations for 24 h. Finally, cell viability was detected according to the manufacturer’s instructions. The percentage of cell viability was calculated using the formula: % cell viability = [(absorbance of the experimental well − absorbance of the blank) / (absorbance of the untreated control well − absorbance of the blank)] × 100. The 50% inhibitory concentration (IC50) was determined from the concentration–response curve.

For EdU assays, 4 × 10^4^ cells per well were seeded into 24-well plates and treated with 50 μM EdU reagent for 2 h at 37 ℃. After fixation and permeabilization, the cells were detected using the Cell-Light EdU Apollo488 Kit (RiboBio, Guangzhou, China) according to the manufacturer’s protocol. Images were captured by LSM880 + 225 Airyscan confocal laser scanning microscope (Carl Zeiss). The proliferation rate was determined by measuring the ratio of EdU-positive cells to DAPI-positive cells.

For the colony formation assay, 1000 cells were plated in 6 cm dishes and cultured for 14–16 days, depending on the growth rate. Colonies were fixed with 4% paraformaldehyde, followed by staining with 0.1% crystal violet (Beyotime) for 20 min. Subsequently, the colonies were photographed and counted.

### Tube formation assay

Cells were plated at a density of 50% confluence into 10 cm dishes. After 48 h of incubation, the medium was replaced with fresh serum-deprived medium, and the cells were cultured for another 24 h. Subsequently, the conditioned medium (CM) was collected, filtered, and concentrated as described previously [23]. For the tube formation assay, 150 µl of Matrigel (Corning, NY, USA) was added to each well of 48-well plates and incubated at 37 °C for 30 min, followed by added HUVECs (4 × 10^4^) in 200 µl of prepared CM to each well and incubated at 37 °C in 5% CO_2_. After 12 h, bright-field images were captured using an inverted microscope and analyzed with WimTube (https://www.wimasis.com/en/WimTube).

### Chicken chorioallantoic membrane (CAM) assay

The CAM assay was performed using day-7 fertilized chicken eggs (Jinan SAIS Poultry Company, Shandong, China) as described previously [26]. Briefly, sterile gelatin sponges mixed with 20 μl of cell suspension containing 4 × 10^6^ cells were deposited on the CAM through a window about 1.0 cm in diameter opened in the eggshell. On embryonic developmental days 12 to 14, the CAMs were harvested, fixated, and photographed. The number of new blood vessels was counted.

### Cytokine arrays and Enzyme-linked immunosorbent assay (ELISA)

Cytokines in the cell supernatant were determined using the G-Series Mouse Inflammation Antibody Array 1 Kit (RayBiotech, GA, USA) by Aksomics Corporation (Shanghai, China) according to the manufacturer’s instructions. Fluorescence signals were collected using a laser scanner, and the raw intensities were revised by background.

For the measurement of cytokine concentrations (IL-6, MIP-1α, and MIG) in the cell supernatant, 1 × 10^6^ cells were cultured in 6 cm dishes with 2 mL serum-free medium for 24 h. The cell suspension was then centrifuged, and the supernatant was filtered with a 0.2 µm filter. Subsequently, the filtered supernatant was subjected to analyze IL-6, MIP-1α, and MIG levels using the corresponding mouse ELISA Kits (Thermo Fisher Scientific) according to the manufacturer’s instructions.

### RNA-sequencing

The total RNA from sgERO1α NTC/T1 null and control cells were isolated using TRIzol Reagent (Invitrogen). Gene expression profiles were analyzed through next-generation sequencing (NGS) with Illumina Nova-Seq by Shanghai Biotechnology Corporation (Shanghai China). mRNAs showing a fold change of 2.0-fold or more (*P* < 0.05) were considered differentially expressed. The list of differentially expressed genes was provided as Supplementary Table S8. Kyoto Encyclopedia of Genes and Genomes (KEGG) pathway enrichment analysis was conducted using Xiantao online tools (https://www.xiantaozi.com/). The KEGG analysis results were visualized using the Lianchuan Cloud platform (https://www.omicstudio.cn/tool/11). The original data are available at NCBI Gene Expression Omnibus (GEO) under accession number GSE246899.

### In-vivo tumor models

BALB/c-nude mice and NOD/SCID mice were obtained from GemPharmatech. Animal experiments were performed according to the guidelines of the Animal Center of Anhui Medical University, and all detailed experimental procedures were approved by the Experimental Animal Ethical Committee of Anhui Medical University.

For cell line-derived xenograft (CDX) assays, 6-week-old male BALB/c-nude mice were randomly assigned into groups (5 mice per group) to receive respective treatments. To observer the in vivo effect of ERO1α and SLC7A11, 4 × 10^6^ corresponding cell lines in 0.2 ml PBS were subcutaneously inoculated into the axilla of mice to establish tumor models. On day 9 after inoculation with shERO1α^1^ LIU-LSC-1 cells, the mice were treated with Lip-1 (10 mg/kg, once every other day, a total of 10 injections) to assess the impact of ferroptosis inhibition on the tumoral growth of ERO1α-knockdown cells. To evaluate the therapeutic efficacy of IKE in CDX models, the mice were treated with IKE (30 mg/kg, once every other day) or vehicle for a total of 10 injections on day 17 after inoculation with sgERO1α NTC/T1 null cells and the control cells.

The LSCC PDX models were established following protocols as described previously [25]. When the passage 3 xenografts reached a mean volume of 100 mm^3^, the mice were randomly divided into 4 groups (*n* = 5 per group), and treated with ERO1α siRNA (100 μg, three times per week) or siNC, together with IKE (40 mg/kg, once every other day) or vehicle.

Lip-1 and IKE were dissolved in vehicle (10% DMSO, 40% PEG300, 5% Tween 80, and 45% saline) and intraperitoneal injection. The chemically modified ERO1α siRNAs (GenePharma) were intratumorally injected. Tumor size was measured every 3 days with a digital caliper, and volume was calculated using the standard formula: volume (mm^3^) = 0.5 × (length × width^2^). Body weights were also monitored. At the endpoint indicated in the corresponding figures, animals were sacrificed, and the subcutaneous tumor masses were taken out for subsequent studies.

### Patient-derived organoid (PDO) model

LSCC organoids were established as described previously with minor modifications [28]. Fresh tumor tissues were washed in ice-cold PBS for 3 × 5 min, and then cut into 1–3 mm^3^ fragments on ice. These fragments were subsequently digested with Trypsin (Sigma-Aldrich) for 30 min at 37 °C. The suspension was collected, filtered through a 100 µm cell filter, and centrifuged at 200 × g for 5 min. The obtained cell clusters were embedded in Matrigel (Corning), and then inoculated into pre-warmed 12-well flat-bottom cell culture plate. After polymerization of the Matrigel balls, 1 ml of HNSCC organoid medium (BioGenous, Suzhou, China) was added, and the medium was renewed approximately every 3–5 days.

For lentiviral infection, the organoids were divided into groups of cells using a pipette. Following centrifugation at 200 × g for 5 min at 4 °C, the cell clusters were suspended in media containing either control lentivirus particles or ERO1α-knockdown lentivirus particles. Subsequently, they were spin-infected in a centrifuge tube (700 × g, 90 min, 25 ℃) and incubated at 37 ℃ for 4 h. Finally, the mixture was centrifuged at 200 × g for 5 min, and the cell clusters were embedded in Matrigel.

For drug test, the indicated LSCC organoids were inoculated into a 96-well cell culture plate at an appropriate density and covered with 100 μL of culture medium. After overnight incubation, the culture medium was replaced with medium containing 50 μM IKE. Twenty-four hours later, the cell viability of the organoids was assessed using the Cell Titer-Glo-3D cell viability assay (Promega) following the manufacture’s instruction.

### Immunohistochemistry (IHC) analysis

The tumor tissues and organoids were fixed in formalin solution, embedded in paraffin, and sliced into thin sections. After dewaxing, rehydrated, and antigen retrieval, the sections were incubated with 3% hydrogen peroxide for 1 h to block endogenous peroxidase activity. Next, after pre-incubation with 3% bovine serum albumin for 1 h, the sections were incubated with primary antibodies at 4 ℃ overnight. The next day, the samples were washed, incubated with HRP-conjugated secondary antibodies, and then developed using DAB solution (Beyotime). Nuclear counterstaining was performed with hematoxylin. A modified histologic score (H-scores [25], [{% of weak staining} × 1] + [{% of moderate staining} × 2] + [{% of strong staining} × 3]) was used for quantitative analysis of IHC staining (Supplementary Fig. 10).

### Bioinformatics analysis

Transcriptome datasets of four types of mTORC1-activated cells and their corresponding control cells were obtained from public channels, including Tsc2 − / − vs. Tsc2 + / + MEFs (https://www.jbc.org/article/S0021-9258(20)44588-0/fulltext#supplementaryMaterial), Tsc1 − / − vs. Tsc1 + / + MEFs (SRP056624), ELT3 (Tsc2-null rat uterine leiomyoma cells) treated with DMSO vs. ELT3 cells treated with rapamycin (GSE183110), and hiPSCs (human induced pluripotent stem cells) Tsc2 − / − vs. hiPSCs Tsc2 + / + cells (GSE171474). The criteria for screening differentially upregulated genes were log2 FC ≥ 1 (or fold change ≥ 2) and *P* < 0.05. The gene lists for all four groups were provided as Supplementary Table S9. We used the online software Venn diagram (https://bioinfogp.cnb.csic.es/tools/venny/) to identify overlapping genes in the four datasets.

### Statistical analysis

Group differences were analyzed using two-tailed student’s t-test or One-way ANOVA as appropriate with GraphPad Prism 6.0 software. A *p*-value < 0.05 was considered significant. *P < 0.05; **P < 0.01; ***P < 0.001; ****P < 0.0001. All data are expressed as the mean ± SD. *P* < 0.05 was defined as statistically significant.

## Results

### Hyperactivated mTORC1 upregulates ERO1α

**Fig. 1:**
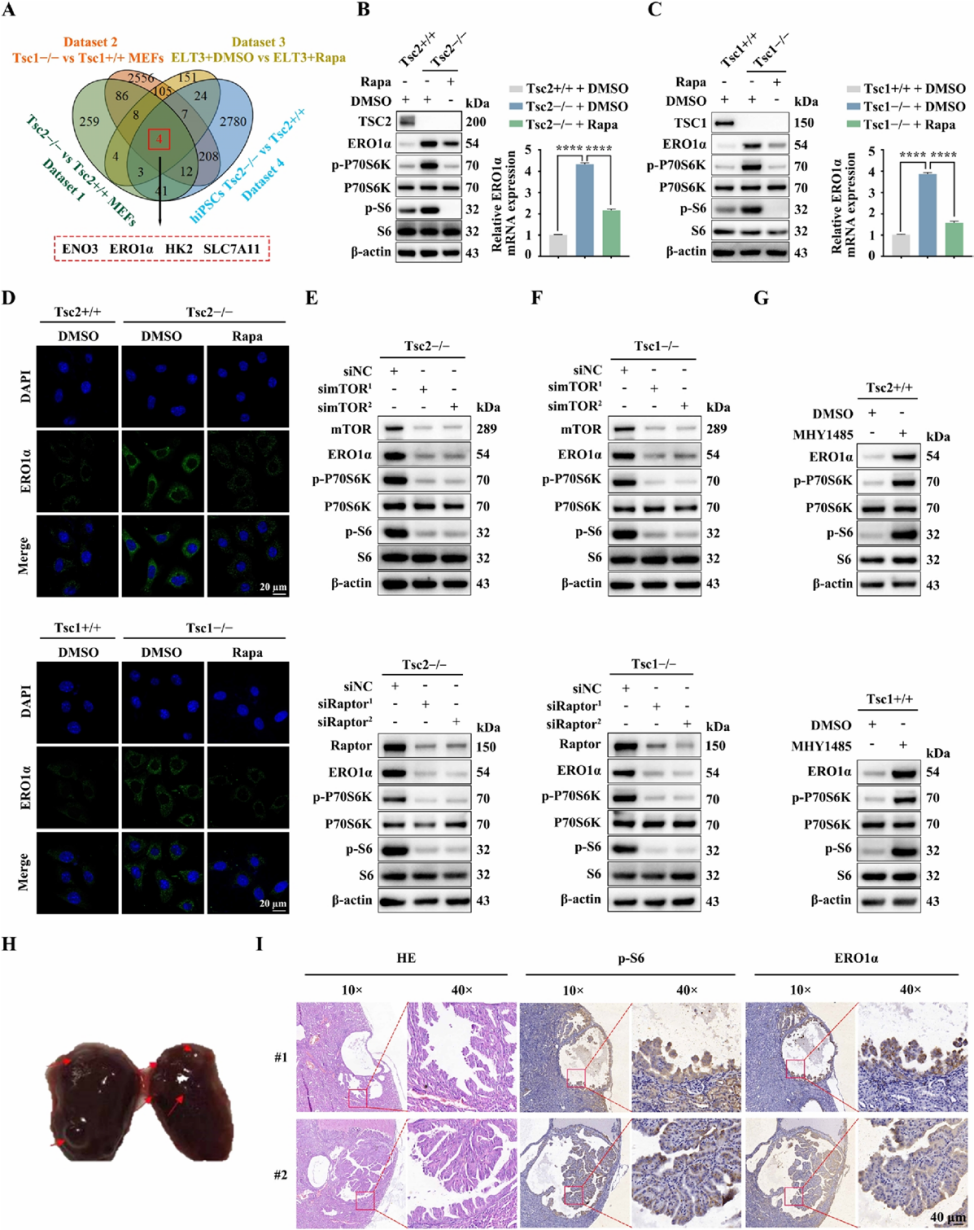
mTORC1 upregulates the expression of ERO1α. **A** Venn diagram analysis of differentially upregulated genes in four datasets. Dataset 1: Tsc2 − / − vs. Tsc2 + / + MEFs. Dataset 2: Tsc1 − / − vs. Tsc1 + / + MEFs. Dataset 3: ELT3 cells treated with DMSO vs. ELT3 cells treated with rapamycin (20 nM, 24 h). Dataset 4: hiPSCs Tsc2 − / − vs. hiPSCs Tsc2 + / + . **B** Tsc2 + / + and Tsc2 − / − MEFs were treated with rapamycin (Rapa, 20 nM) or DMSO for 24 h. **C** Tsc1 + / + and Tsc1 − / − MEFs were treated with rapamycin (Rapa, 20 nM) or DMSO for 24 h. **B** and **C** Cell lysates were subjected to immunoblotting with the indicated antibodies (left panels); ERO1α levels were analyzed by qRT–PCR (right panels). **D** IF analysis of the expression of ERO1α in the indicated cells. Scale bar, 20 μm. **E** and **F** Tsc2 − / − (E) or Tsc1 − / − (F) MEFs were transfected with siRNA targeting mTOR (simTOR), Raptor (siRaptor) or the control (siNC) for 48 h. **G** Tsc2 + / + or Tsc1 + / + MEFs were treated with 5 μM MHY1485 for 24 h. **E**–**G** Cell lysates were subjected to immunoblotting with the indicated antibodies. **H** A representative kidney of Tsc2 + / − mice. Red arrows indicate renal cysts and cystadenomas. **I** Representative IHC images of p-S6 and ERO1α staining from renal cystadenomas of Tsc2 + / − mice. Error bars indicate mean ± SD of triplicate samples. ****P < 0.0001

Because the TSC1/TSC2 complex is the principal suppressor of mTORC1, Tsc1 or Tsc2-null cells are good models for studying mTORC1 signaling. To identify the functional genes associated with mTORC1 activation across species, we created a Venn diagram using differentially upregulated genes from four transcriptome profiling datasets, including Tsc2 − / − vs. Tsc2 + / + MEFs, Tsc1 − / − vs. Tsc1 + / + MEFs, ELT3 treated with DMSO vs. ELT3 cells treated with rapamycin, and hiPSCs Tsc2 − / − vs. hiPSCs Tsc2 + / + cells [29–32]. Four overlapping upregulated genes were screened, including enolase 3 (*ENO3*), *ERO1α*, hexokinase (*HK2*), and *SLC7A11* (Fig. 1A). We have previously reported that mTORC1 suppresses melanin synthesis and promotes tumorigenesis through SLC7A11 upregulation [33]. HK2 and ENO3 are representative glycolytic enzymes, and it is well recognized that mTORC1 signaling promotes glycolysis [34–36], whereas the function of ERO1α in mTORC1-activated cells remains unclear. Subsequently, western blot and qRT − PCR analyses confirmed that ERO1α expression was significantly increased in Tsc1- or Tsc2-null MEFs compared to control cells, and its level was reversed by mTORC1 inhibition with rapamycin treatment (Fig. 1B, C), which was further verified by IF staining (Fig. 1D).

To further substantiate that it is indeed mTORC1 that mediates the positive regulation of ERO1α downstream of the TSC1/TSC2 complex, we detected ERO1α levels in mTOR, Raptor (a specific component of mTORC1) or Rictor (a specific component of mTORC2) knockdown Tsc2 − / − MEFs. As shown in Fig. 1E and Supplementary Fig. 1A, cells transfected with mTOR or Raptor siRNAs exhibited significantly decreased ERO1α levels, while transfection with Rictor siRNAs had little effect on ERO1α expression. A similar result was obtained in Tsc1 − / − MEFs after either knockdown of mTOR, Raptor or Rictor (Fig. 1F, Supplementary Fig. 1B). In contrast, activation of mTORC1 upon treatment of MHY1485 led to upregulation of ERO1α in Tsc2 + / + or Tsc1 + / + MEFs (Fig. 1G). In addition, consistent with the expression of p-S6 (an indicator of mTORC1 activity), the expression of ERO1α was elevated in the renal cystadenomas of Tsc2 + / − mice (Fig. 1H, I). Together, these data suggest that hyperactivated mTORC1 upregulates ERO1α expression.

*ERO1α* is a hypoxia-responsive gene, and HIF-1α is a well-known target of mTORC1 [37, 38]. Hence, HIF-1α may be a candidate transcription factor involved in mTORC1-mediated upregulation of ERO1α. As expected, the knockdown of HIF-1α led to marked downregulation of ERO1α in Tsc2 − / − or Tsc1 − / − MEFs (Supplementary Fig. 2A, B). Moreover, silencing of HIF-1α suppressed hypoxia-induced (DFX) ERO1α upregulation in Tsc2 + / + or Tsc1 + / + MEFs (Supplementary Fig. 2C, D). Overall, mTORC1 promotes ERO1α expression through HIF-1α upregulation.

### ERO1α promotes cell proliferation, angiogenesis, and tumor growth

**Fig. 2:**
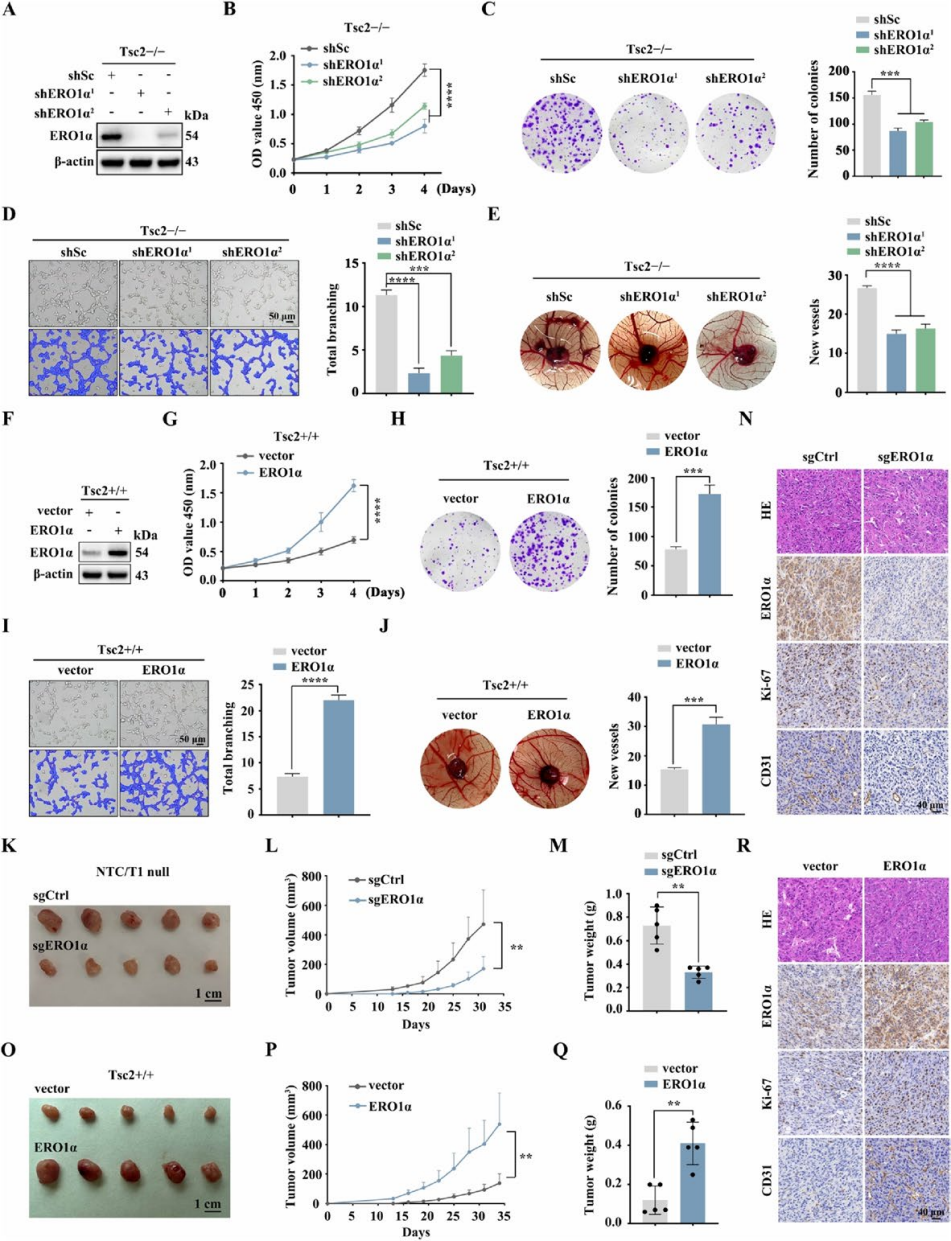
ERO1α promotes cell proliferation, angiogenesis, and tumor growth driven by mTORC1 activation. **A**–**E** Tsc2 − / − MEFs were transduced with lentivirus expressing shRNAs against ERO1α (shERO1α^1^ and shERO1α.^2^) or a scrambled sequence (shSc). **F**–**J** Tsc2 + / + MEFs were infected with control (vector) lentiviruses or lentiviruses encoding ERO1α. **A**–**J** The expression of ERO1α was assessed by western blotting (**A** and **F**); the cell proliferation was evaluated by CCK-8 (**B** and **G**) and colony formation (**C** and **H**) assays; the effect on angiogenesis was determined by tube formation (**D** and **I**) and CAM assays (**E** and **J**). Representative images (left panels) and quantifications (right panels). **K**–**R** Tumor growth of mice subcutaneously inoculated with the indicated cells. *N* = 5 for each group. **K** and **O** Tumor pictures. **L** and **P** Tumor growth curves. **M** and **Q** Tumor weight. **N** and **R** Representative IHC staining for ERO1α, Ki-67, and CD31 of the indicated tumor tissues. Scale bar, 40 μm. Error bars indicate mean ± SD of triplicate (if mentioned otherwise) samples. **P < 0.01; ***P < 0.001; ****P < 0.0001

To clarify the biological functions of ERO1α in mTORC1-activated cells, we silenced ERO1α in Tsc2-deficient MEFs using two short hairpin RNAs (shRNAs), designated here as shERO1α^1^ and shERO1α^2^. Both these ERO1α-specific shRNAs efficiently suppressed ERO1α expression compared to the scrambled control shRNA (Fig. 2A). CCK-8 and colony formation assays showed that knockdown of ERO1α significantly reduced the proliferation and colony formation ability of Tsc2 − / − MEFs (Fig. 2B, C). Moreover, an in vitro capillary tube formation assay was used to evaluate the angiogenesis activities of ERO1α. HUVECs cultured with a conditioned medium derived from ERO1α knockdown cells developed fewer capillary-like structures and branch points, suggesting the pro-angiogenic effect of ERO1α (Fig. 2D). This was further proven by a CAM assay, which revealed that knockdown of ERO1α strongly ablated the formation of new micro-vessels (Fig. 2E). In addition, ERO1α was ectopically overexpressed in Tsc2 + / + MEFs (Fig. 2F). In contrast to knockdown of ERO1α, ERO1α overexpression promoted cell proliferation, colony formation, and enhanced the angiogenic capacity of Tsc2 + / + MEFs (Fig. 2G–J). In summary, these findings suggest that ERO1α could promote the proliferation and enhance the pro-angiogenic capacity of mTORC1-activated cells.

Because Tsc1 − / − or Tsc2 − / − MEFs have low tumorigenic ability in vivo [39], we constructed a novel cell line (NTC/T1 null cells) with potent tumorigenicity derived from a subcutaneous tumor formed by the injection of Tsc1 − / − MEFs into nude mice to investigate the in vivo role of ERO1α (Supplementary Fig. 3A–E). ERO1α was knocked out in NTC/T1 null cells by transfecting with a CRISPR/Cas9 sgRNA lentiviral construct (Supplementary Fig. 3F). As shown in Supplementary Fig. 3G and H, ERO1α deletion substantially suppressed the proliferative and angiogenic abilities of NTC/T1 null cells. Subsequently, we performed a xenograft assay by subcutaneous injection of sgERO1α NTC/T1 null cells and control (sgCtrl) cells into the right anterior armpits of nude mice. The mice injected with sgERO1α cells had significantly reduced tumor volume and weight compared to those injected with sgCtrl cells (Fig. 2K–M). Furthermore, IHC analysis of mouse xenograft tumor tissues demonstrated a significant reduction in cell proliferative marker Ki-67 and angiogenic marker CD31 levels in sgERO1α tumors relative to the control counterpart (Fig. 2N). In contrast, the results of a xenograft assay with ERO1α-overexpressing Tsc2 + / + MEFs showed that overexpression of ERO1α promotes tumor cell proliferation and angiogenesis in vivo (Fig. 2O–R). Therefore, ERO1α positively regulates angiogenesis and tumor growth driven by mTORC1 activation.

### Elevated ERO1α promotes ferroptosis resistance driven by mTORC1 activation

**Fig. 3:**
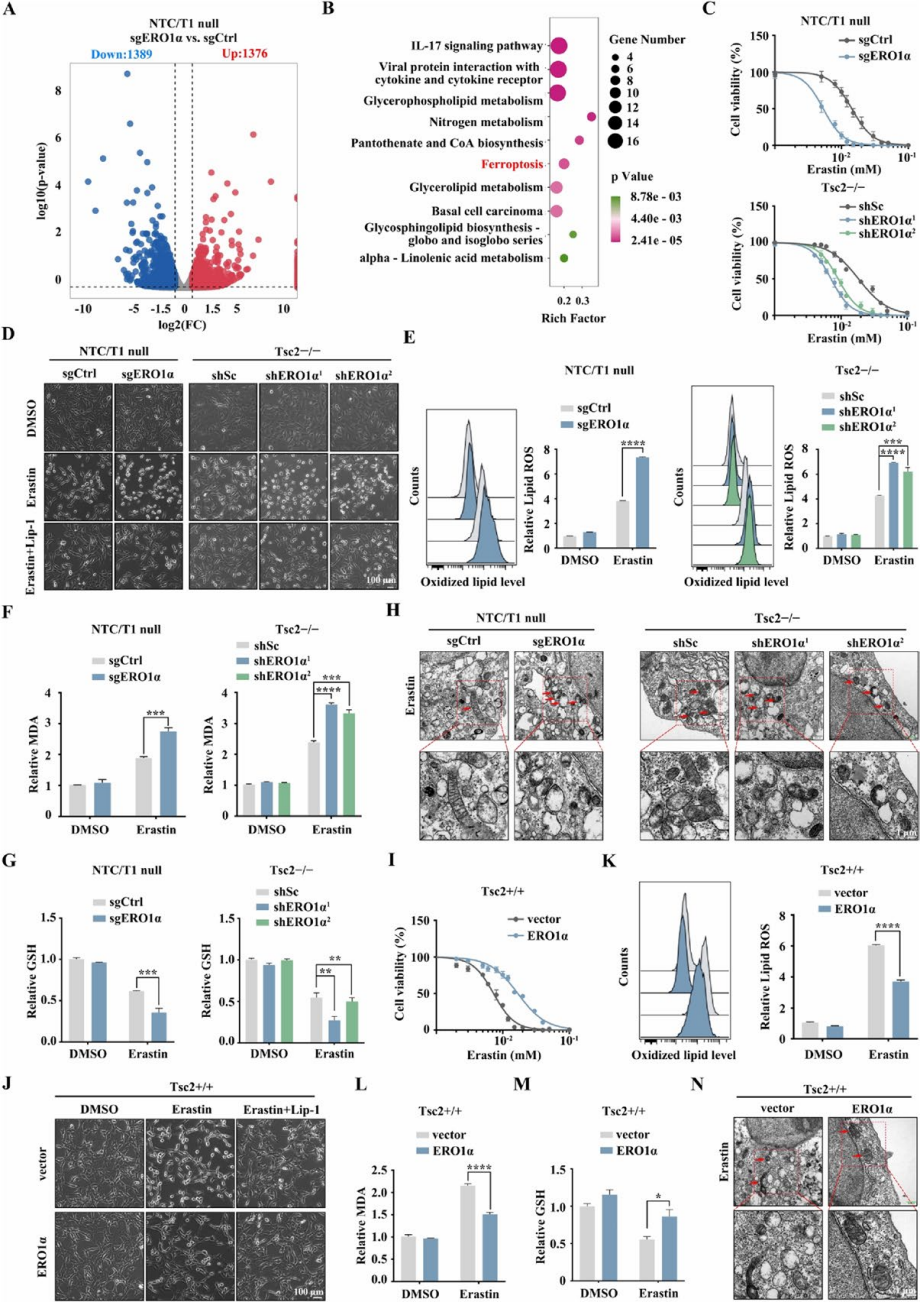
ERO1α facilitates resistance to ferroptosis. **A** and **B** sgERO1α and sgCtrl NTC/T1 null cells were subjected to RNA-seq analysis. **A** Volcano plot of differentially expressed genes. **B** KEGG enrichment analysis of differentially downregulated genes. **C** and **I** Cell viability of the indicated cells following treatment with erastin for 24 h. **D** and **J** The indicated cells were treated with or without erastin (10 μM) in the absence or presence of Lip-1 (1 μM) for 24 h. The corresponding phase contrast images are shown. Scale bar, 100 μm. **E**–**G** and **K**–**M** The indicated cells were treated with or without erastin (10 μM) for 24 h, and then L-ROS (**E** and **K**), intracellular MDA (**F** and **L**), and intracellular GSH (**G** and **M**) were assayed. **H** and **N** Representative TEM images of the mitochondrial morphology in the indicated cells treated with 10 μM erastin for 16 h. Red arrows indicate mitochondria. Scale bar, 1 μm. Error bars indicate mean ± SD of triplicate samples. *P < 0.05; **P < 0.01; ***P < 0.001; ****P < 0.0001

RNA sequencing analysis identified a total 2765 differentially expressed genes (DEGs), including 1376 upregulated genes and 1389 downregulated genes, in sgERO1α NTC/T1 null cells compared to control cells (Fig. 3A). KEGG pathway analysis for the differentially downregulated genes revealed that the ferroptosis pathway was present among the top 10 dysregulated pathways (Fig. 3B). It has been known that mTORC1 promotes ferroptosis resistance [13]. Indeed, compared to the control cells, Tsc2- or Tsc1-null MEFs were more resistant to erastin-induced lipid peroxidation and cell death, and this effect could be almost entirely reversed by rapamycin treatment (Supplementary Fig. 4A–E). To verify whether ERO1α is involved in mTORC1-mediated ferroptosis resistance, we tested sgERO1α NTC/T1 null cells and the control cells to uncover drug sensitivity with erastin. As shown in the upper panel of Fig. 3C, knockout of ERO1α increased the sensitivity of NTC/T1 null cells to erastin. Similarly, the knockdown of ERO1α also sensitized erastin to growth inhibition in Tsc2 − / − MEFs (Fig. 3C, lower panel). We also observed that knockout or knockdown of ERO1α aggravated erastin-induced cell death in NTC/T1 null cells or Tsc2 − / − MEFs (Fig. 3D). Notably, erastin-induced cell death could be rescued by ferroptosis inhibitor Lip-1 (Fig. 3D), which further indicates the occurrence of ferroptosis. We next used BODIPY-C11 (a lipid-soluble ratiometric fluorescent indicator of lipid peroxidation) to estimate the level of ferroptosis-associated lipid peroxidation in ERO1α knockout or knockdown cells. As represented in Fig. 3E, the depletion of ERO1α exacerbated erastin-induced lipid peroxidation. Accordingly, ERO1α silencing also enhanced erastin-induced accumulation of intracellular MDA, which is one of the final products of lipid peroxidation (Fig. 3F). Considering that the loss of cell redox balance causes ferroptosis, GSH plays vital role in eliminating the accumulation of lipid ROS. We next investigated whether ERO1α depletion is related to GSH synthesis. As shown in Fig. 3G, the GSH levels were significantly suppressed upon ERO1α knockout or knockdown. Moreover, transmission electron microscopy results showed that ERO1α knockout or knockdown cells treated with erastin displayed shrunken mitochondria and increased membrane density, a hallmark of ferroptosis. However, these phenotypes were alleviated in control cells after erastin treatment (Fig. 3H). This further indicated that depletion of ERO1α promoted ferroptosis in mTORC1-activated cells.

To further examine whether ERO1α induces resistance to ferroptosis, we tested ERO1α-overexpressing Tsc2 + / + MEFs and the control cells for sensitivity to erastin. As shown in Fig. 3I and J, cells with ectopic expression of ERO1α were resistant to erastin-induced cell death. Moreover, overexpression of ERO1α decreased erastin-induced elevation of the peroxide levels and GSH depletion (Fig. 3K–M). Correspondingly, erastin-mediated morphological manifestations related to ferroptosis in mitochondria could be largely reversed by ERO1α overexpression (Fig. 3N). Our results demonstrate that elevated ERO1α stimulated ferroptosis resistance in mTORC1-activated cells.

### ERO1α promotes ferroptosis resistance and tumor progression via upregulation of SLC7A11

**Fig. 4:**
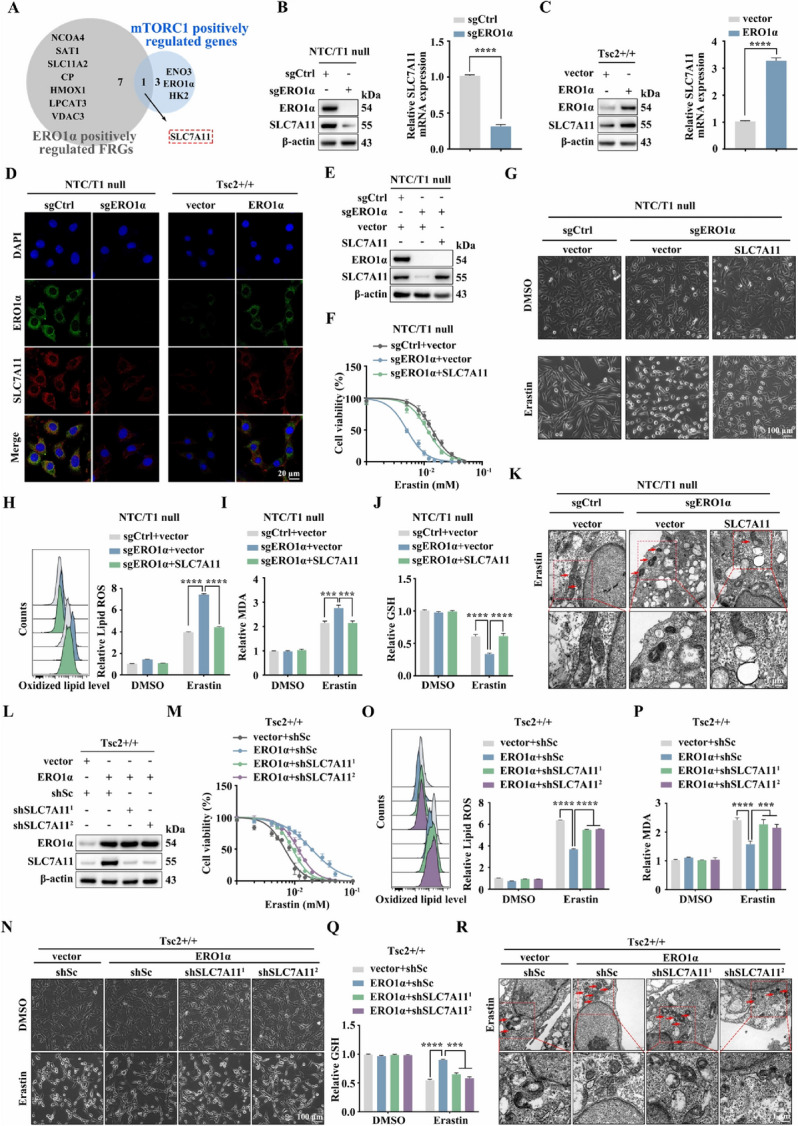
ERO1α facilitates ferroptosis resistance through the upregulation of SLC7A11. **A** Venn diagram of the ferroptosis-related genes (FRGs) positively regulated by ERO1α and mTORC1 positively regulated genes. **B** sgERO1α and sgCtrl NTC/T1 null cells. **C** ERO1α-overexpressing Tsc2 + / + MEFs and the control cells. **B** and **C** SLC7A11 levels were examined by western blotting (left panels) and qRT–PCR (right panels). **D** IF analysis of the expression of SLC7A11 in the indicated cells. Scale bar, 20 μm. **E**–**K** sgERO1α NTC/T1 null cells were infected with lentiviruses carrying an empty vector (vector) or expression vectors for SLC7A11. **L**–**R** ERO1α-expressing Tsc2 + / + MEFs were infected with lentivirus harboring SLC7A11 shRNAs (shSLC7A11^1^ and shSLC7A11.^2^) or a scrambled shRNA (shSc). **E** and **L** SLC7A11 and ERO1α protein levels were examined by western blotting. **F** and **M** Cell viability was assessed after treatment with different concentrations of erastin for 24 h in the indicated cells. **G** and **N** Representative phase-contrast images of the indicated cells treated with erastin (10 μM, 24 h) or DMSO. Scale bar, 100 μm. **H**–**J** and **O**–**Q** The indicated cells were treated with or without erastin (10 μM) for 24 h, and then L-ROS (**H** and **O**), intracellular MDA (**I** and **P**), and intracellular GSH (**J** and **Q**) were measured. **K** and **R** Representative TEM images of the indicated cells treated with 10 μM erastin for 16 h. Red arrows indicate mitochondria. Scale bar, 1 μm. Error bars indicate mean ± SD of triplicate samples. ***P < 0.001; ****P < 0.0001

Since ERO1α mediates ferroptosis resistance and tumor growth induced by mTORC1 activation, our next goal is to search for downstream effectors of the mTORC1/ERO1α pathway. Therefore, a Venn diagram analysis on the ferroptosis-related genes (FRGs) positively regulated by ERO1α (Fig. 3B) and mTORC1 positively regulated genes (Fig. 1A) was performed. SLC7A11, a critical ferroptosis regulator and tumor promoter, was screened out (Fig. 4A). Western blot and qRT − PCR analyses confirmed the inhibition of SLC7A11 when ERO1α knockout was present at both the mRNA and protein levels (Fig. 4B). A consistent result was obtained in Tsc2 − / − MEFs with knockdown of ERO1α (Supplementary Fig. 5A, B). In contrast, ectopic expression of ERO1α led to the upregulation of SLC7A11 (Fig. 4C). IF staining confirmed the positive regulatory effect of ERO1α on SLC7A11 expression (Fig. 4D).

To determine whether the downregulation of SLC7A11 is essential for ERO1α loss-induced ferroptosis, we re-expressed SLC7A11 in sgERO1α NTC/T1 null cells by transfecting the *SLC7A11* gene (Fig. 4E). The forced expression of SLC7A11 restored ferroptosis resistance in sgERO1α cells, which was related to decreased cell death, oxidative damage (L-ROS and MDA), and GSH depletion (Fig. 4F–J). Notably, restoration of SLC7A11 in sgERO1α cells mostly suppressed erastin-triggered ferroptosis mitochondrial changes, such as shrunken mitochondria and fewer mitochondrial crista (Fig. 4K). Similar to sgERO1α NTC/T1 null cells, re-expression of SLC7A11 in shERO1α^1^ Tsc2 − / − MEFs inhibited lipid peroxidation and reversed the enhanced sensitivity to erastin upon knockdown of ERO1α (Supplementary Fig. 5C–H). To further explicate the effect of SCL7A11 in ERO1α-mediated ferroptosis resistance, we silenced endogenous SLC7A11 by transducing lentiviral vectors expressing shRNA for SLC7A11 to ERO1α-overexpressing Tsc2 + / + MEFs. The knockdown efficiency was confirmed by western blotting (Fig. 4L). As expected, SLC7A11 knockdown reversed the resistance of ERO1α-overexpressing Tsc2 + / + MEFs to erastin-induced growth inhibition, cell death, lipid peroxidation, GSH downregulation, and changes in mitochondrial morphology (Fig. 4M–R).

**Fig. 5:**
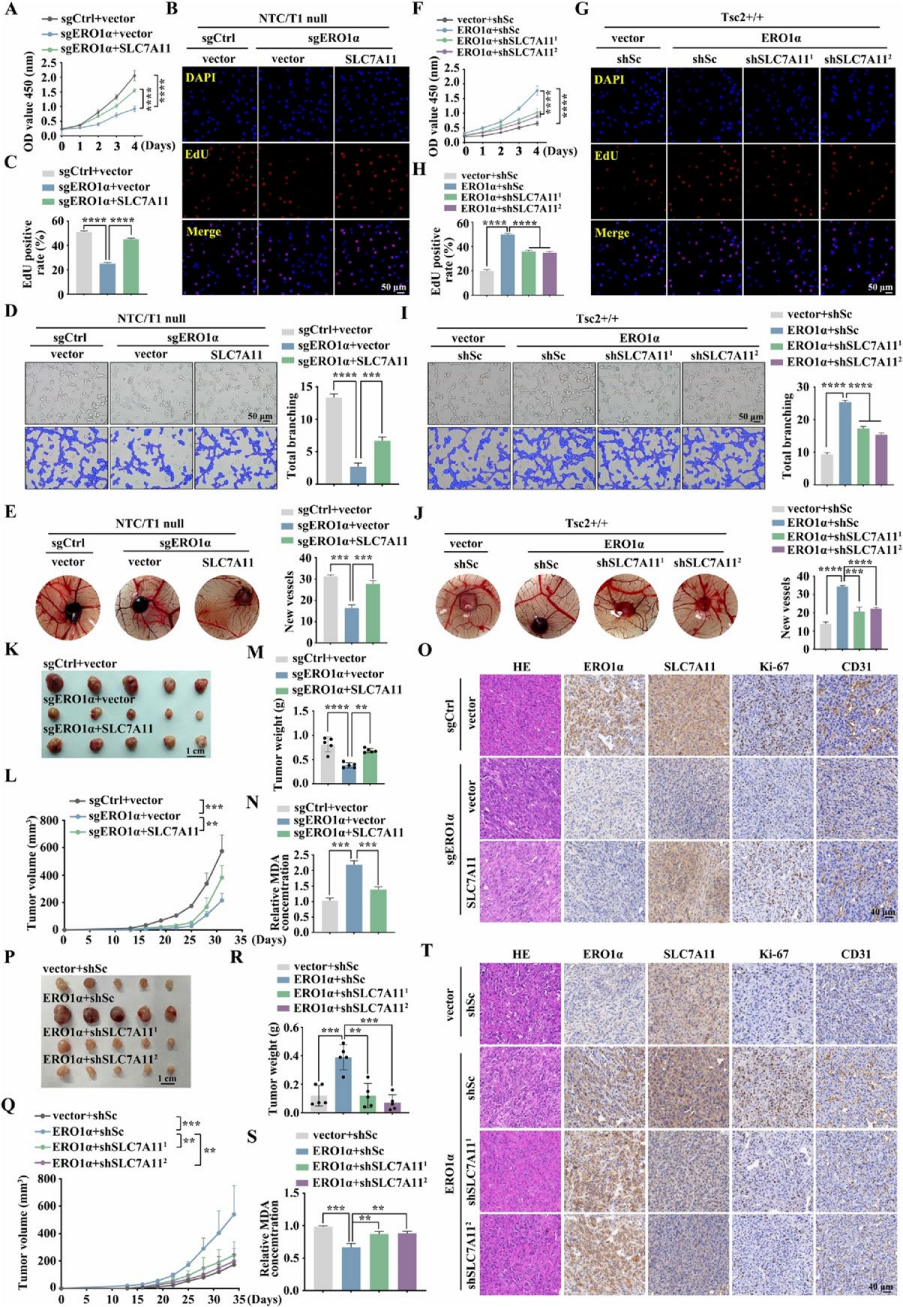
ERO1α exhibits tumor-promoter activities through the upregulation of SLC7A11. **A**–**E** sgERO1α NTC/T1 null cells were infected with lentiviruses carrying an empty vector (vector) or expression vectors for SLC7A11. **F**–**J** ERO1α-overexpressing Tsc2 + / + MEFs were infected with lentivirus harboring SLC7A11 shRNAs (shSLC7A11^1^ and shSLC7A11.^2^) or a scrambled shRNA (shSc). CCK-8 (**A** and **F**) and EdU (**B**, **C**, **G** and **H**) assays were performed to evaluate cell proliferation. Scale bar, 50 μm. The effect on angiogenesis was determined by tube formation (**D** and **I**) and CAM (**E** and **J**) assays. Representative images (left panels) and quantifications (right panels). Scale bar, 50 μm. **K**–**T** sgERO1α NTC/T1 null cells, with or without SLC7A11 re-expression (**K**–**O**), and ERO1α-overexpressing Tsc2 + / + MEFs, with or without SLC7A11 knockdown (**P**–**T**), were subcutaneously injected into nude mice for xenograft assays. **K** and **P** Pictures of the removed tumors. **L** and **Q** The size of xenograft tumors was measured. **M** and **R** Tumors were weighed and plotted. **N** and **S** The relative MDA levels of the indicated tumors were measured. **O** and **T** Representative IHC images for ERO1α, SLC7A11, Ki-67, and CD31 proteins of the indicated xenograft tumors. Scale bar, 40 μm. Error bars indicate mean ± SD of triplicate (if mentioned otherwise) samples. **P < 0.01; ***P < 0.001; ****P < 0.0001

Next, we examined whether the pro-oncogenic effect of ERO1α in mTORC1-activated cells depends on SLC7A11. As shown in Fig. 5A, restoration of SLC7A11 in sgERO1α cells partially rescued ERO1α knockout–induced suppression of cell growth. Moreover, the proliferative ability of sgERO1α cells was significantly promoted by SLC7A11 re-expression, as determined by EdU staining (Fig. 5B, C). Furthermore, tube formation and CAM assays demonstrated that SLC7A11 overexpression attenuated the inhibitory effect of ERO1α deficiency on angiogenesis (Fig. 5D, E). Consistent with sgERO1α NTC/T1 null cells, restoration of SLC7A11 efficiently reversed the adverse effects of ERO1α knockdown on the proliferative and angiogenic abilities of Tsc2 − / − MEFs (Supplementary Fig. 6A–D). In contrast, the accelerated cell proliferation and angiogenesis induced by ERO1α overexpression were inhibited by knockdown of SLC7A11 in Tsc2 + / + MEFs (Fig. 5F–J). Overall, these data underscore the importance of ERO1α-regulated SLC7A11 expression in the tumorigenesis of mTORC1-activated cells.

The mediation effect of SLC7A11 on ERO1α-regulated cell growth and angiogenesis was further verified in vivo using a subcutaneous xenograft tumor model. Consistent with the in vitro results, SLC7A11 re-expression abrogated the suppressive effects of ERO1α depletion on xenograft growth, tumor cell proliferation (as assessed by the Ki-67 index), and tumor angiogenesis (as assessed by the CD31 index) (Fig. 5K–O). Conversely, ERO1α overexpression-induced xenograft tumor development and ferroptosis inhibition were significantly attenuated with suppression of SLC7A11 (Fig. 5P–T). Our results indicate that ERO1α promotes tumor progression and ferroptosis resistance, at least partially, by upregulating SLC7A11.

### ERO1α upregulates SLC7A11 via activation of the IL-6/STAT3 pathway

**Fig. 6:**
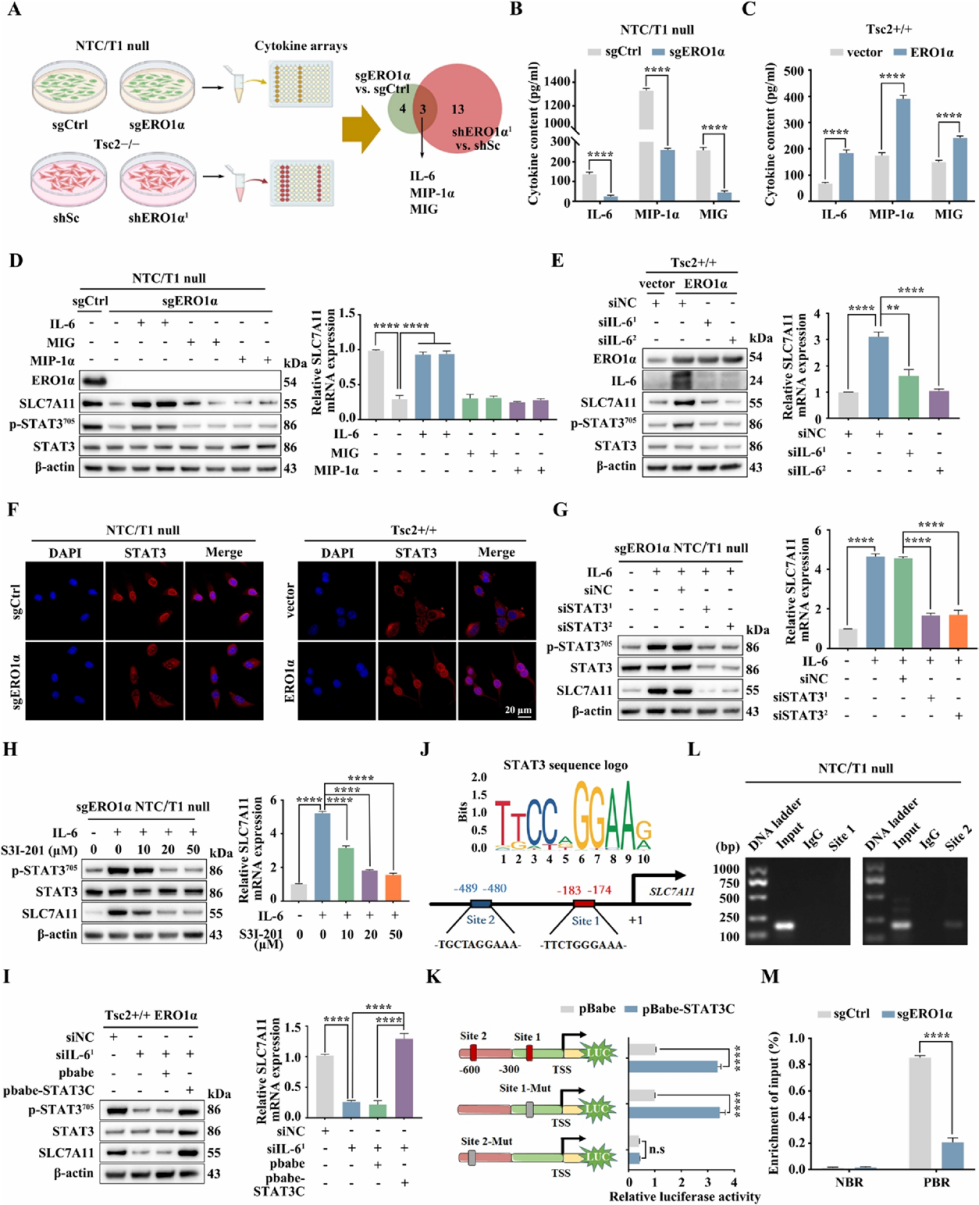
ERO1α upregulates SLC7A11 via activation of the IL-6/STAT3 pathway. **A** Schematic diagram of the screening of co-differentially expressed cytokines upon knockout or knockdown of ERO1α using a cytokines array assay. **B** and **C** Cell supernatants from the indicated cells were collected, and IL-6, MIP-1α, and MIG levels were determined using an ELISA. **D** sgERO1α NTC/T1 null cells were treated with IL-6 (20 ng/ml), MIP-1α (100 ng/ml), or MIG (100 ng/ml) for 24 h. **E** ERO1α-overexpressing Tsc2 + / + MEFs were transfected with IL-6 siRNAs or control siRNA (siNC) for 48 h. **D** and **E** Cell lysates were subjected to immunoblotting with the indicated antibodies (left panels), the expression of SLC7A11 mRNA was detected by qRT–PCR (right panels). **F** Representative IF showing the localizations of STAT3 in the indicated cells. Scale bar, 20 μm. **G** IL-6 (20 ng/ml, 12 h) pre-treated sgERO1α NTC/T1 null cells were transfected with STAT3 siRNAs or control siRNA (siNC) for 48 h. **H** IL-6 (20 ng/ml, 12 h) pre-treated sgERO1α NTC/T1 null cells were treated with different concentrations of S3I-201 for 24 h. **I** IL-6 siRNA-transduced ERO1α-overexpressing Tsc2 + / + MEFs were transfected with a constitutively activated STAT3 (STAT3C) or its control vector pBabe-puro (pBabe). **G**–**I** The expression of SLC7A11 was examined by western blotting (left panels) and qRT–PCR (right panels). **J** Schematic representation of the putative STAT3-binding sites in the promoter of mouse *SLC7A11* gene. **K** HEK 293 T cells were co-transfected with the indicated promoter constructs plus pBabe-STAT3C or empty vector pBabe and the internal control plasmid pRL-TK. The relative luciferase activity was determined 24 h after transfection. **L** The enrichment of STAT3 in the promoter of *SLC7A11* was analyzed by ChIP-PCR assay. **M** sgERO1α and sgCtrl NTC/T1 null cells were subjected to ChIP analysis with antibodies to p-STAT3 or control rabbit IgG. qRT–PCR was performed to amplify regions surrounding the putative STAT3 binding Site 2 (PBR) and a nonspecific STAT3 binding region (NBR). The data were plotted as the ratio of immunoprecipitated DNA to total input DNA. Error bars indicate mean ± SD of triplicate samples. **P < 0.01; ****P < 0.0001. n.s: no significance

As a key enzyme for disulfide bond formation, ERO1α plays a critical role in the folding process of the new peptide chain to form a functional secreted protein [40, 41]. Therefore, we next collected the conditioned media from sgERO1α NTC/T1 null cells, shERO1α^1^ Tsc2 − / − MEFs, and their corresponding control cells and conducted a cytokine array assay (Fig. 6A). Results showed that three cytokines, including IL-6, MIP-1α, and MIG, were significantly decreased upon knockout and knockdown of ERO1α in mTORC1-activated cells (Supplementary Fig. 7A). ELISA assays confirmed that ERO1α depletion markedly downregulated the levels of secreted IL-6, MIP-1α, and MIG in NTC/T1 null cells and Tsc2 − / − MEFs. However, overexpression of ERO1α promoted the expression of these cytokines (Fig. 6B, C, Supplementary Fig. 7B). IL-6 treatment rescued the decreased levels of SLC7A11 induced by knockout or knockdown of ERO1α. In contrast, administration of MIP-1α and MIG had little effect on the expression of SLC7A11 in ERO1α knockout or knockdown cells (Fig. 6D, Supplementary Fig. 7C). Therefore, ERO1α likely promotes the expression of SLC7A11 by upregulating of IL-6. In line with this view, the knockdown of IL-6 abrogated the overexpression of ERO1α-induced upregulation of SLC7A11 in Tsc2 + / + MEFs (Fig. 6E). It is well known that IL-6 drives oncogenic activation of STAT3 in multiple cells [42]. Interestingly, IL-6 treatment reversed the attenuated STAT3 activity driven by depletion of ERO1α in NTC/T1 null or Tsc2 − / − MEFs, while knockdown of IL-6 attenuated the enhanced STAT3 activity due to ERO1α overexpression in Tsc2 + / + MEFs (Fig. 6D, E, Supplementary Fig. 7C). IF analysis also confirmed that the nuclear accumulation of STAT3 was positively regulated by ERO1α (Fig. 6F, Supplementary Fig. 7D). Moreover, inhibition of STAT3 by genetic or pharmacologic strategies suppressed IL-6-induced upregulation of SLC7A11 in sgERO1α NTC/T1 null cells (Fig. 6G, H). A similar result was also observed in ERO1α-KD Tsc2–/– MEFs (Supplementary Fig. 7E, F). On the contrary, overexpression of the constitutively activated form of STAT3 (STAT3C) significantly compromised the inhibitory effect of IL-6 knockdown on SLC7A11 expression in ERO1α-overexpressing Tsc2 + / + MEFs (Fig. 6I). Hence, we propose that ERO1α stimulates SLC7A11 expression via activation of the IL-6/STAT3 pathway.

To further investigate the mechanisms underlying STAT3 regulation of SLC7A11, we analyzed the potential upstream promoter region (− 2000 to + 200) of mouse SLC7A11 using JASPAR (http://jaspar.genereg.net), which predicted two possible binding regions of STAT3 (Fig. 6J). We then cloned the mouse *SLC7A11* gene promoter (− 575 to + 144) into the pGL3 luciferase reporter vector and evaluated the effect of STAT3 on the promoter activity. The promoter activity of the wild-type SLC7A11 construct was enhanced by STAT3C overexpression. Notably, mutation of Site 2 completely abolished the stimulatory effect of STAT3C, whereas mutation of Site 1 showed only a slight effect on STAT3C-induced SLC7A11 promotor activity (Fig. 6K). The ChIP-PCR assay indicated that STAT3 was only enriched at Site 2, further suggesting that this site is critically important for the transcription of SLC7A11 (Fig. 6L). Moreover, the ChIP − qRT − PCR assay revealed decreased occupancy of STAT3 on the *SLC7A11* promoter in sgERO1α cells and increased occupancy in ERO1α-overexpressing cells (Fig. 6M, Supplementary Fig. 7G). Therefore, we conclude that activated STAT3 transcriptionally elevates SLC7A11 by directly binding to its promoter in response to the ERO1α/IL-6 pathway activation.

### The mTORC1/ERO1α/IL-6/STAT3/SLC7A11 signaling pathway presents in human cancer

**Fig. 7:**
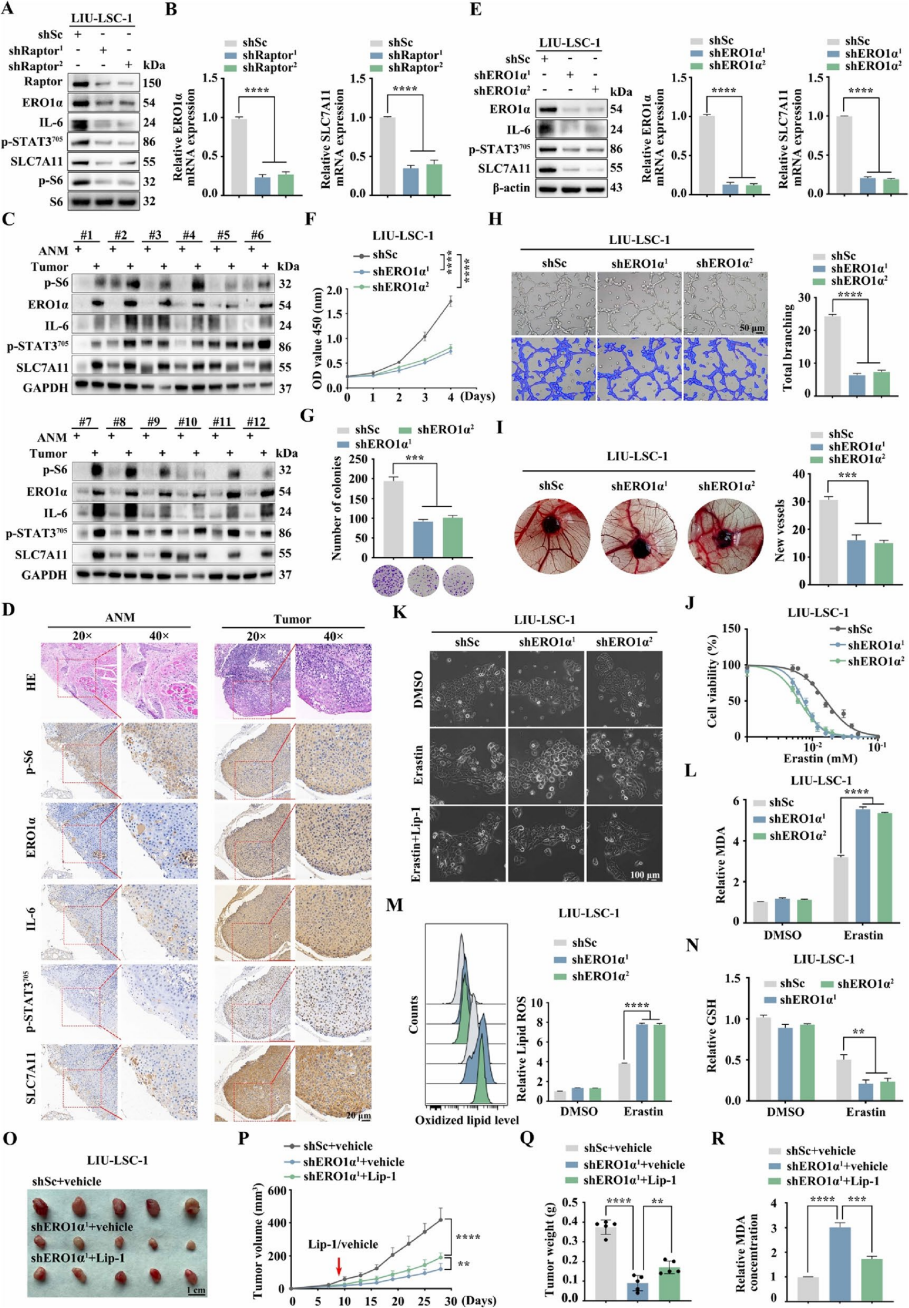
The mTORC1/ERO1α/IL-6/STAT3/SLC7A11 signaling pathway exists in human cancer. **A** and **B** LIU-LSC-1 cells were infected with lentivirus expressing shRNAs targeting Raptor (shRaptor^1^ and shRapator^2^) or a control shRNA (shSc). Cell lysates were subjected to immunoblotting with the indicated antibodies (**A**), ERO1α and SLC7A11 mRNA levels were detected by qRT–PCR (**B**). **C** 12 paired LSCC tissues and the corresponding ANM tissues were subjected to immunoblotting with the indicated antibodies. **D** Representative IHC images of p-S6, ERO1α, IL-6, p-STAT3, and SLC7A11 staining from the LSCC tissues and ANM tissues. Scale bar, 20 μm. **E**–**I** LIU-LSC-1 were infected with lentiviruses expressing shRNAs targeting ERO1α (shERO1α^1^ and shERO1α^2^) or a non-targeting shRNA (shSc). **E** The protein and mRNA levels of ERO1α and SLC7A11 were determined by western blotting and qRT–PCR. **F** and **G** CCK-8 (**F**) and colony formation (**G**) assays were performed to evaluate cell growth. **H** and **I** The pro-angiogenic effect of ERO1α was detected by tube formation (**H**) and CAM assays (**I**). Representative images (left panels) and quantifications (right panels) are shown. **J** Cell viability of indicated cells following treatment with erastin for 24 h. **K** Representative phase-contrast images of indicated cells were treated with erastin (15 μM) in the absence or presence of Lip-1 (1 μM). The corresponding phase contrast images are shown. Scale bar, 100 μm. **L**–**N** The indicated cells were treated with or without erastin (15 μM) for 24 h, and then intracellular MDA (**L**), L-ROS (**M**), and intracellular GSH (**N**) were measured. **O**–**R** Tumor images (**O**), tumor volume (**P**), and tumor weight (**Q**) of shERO1α.^1^ LIU-LSC-1 xenograft tumors (*n* = 5 mice/group) treated with Lip-1 (10 mg/kg) or vehicle. **R** The relative MDA levels of the indicated tumors were measured. Error bars indicate mean ± SD of triplicate (if mentioned otherwise) samples. **P < 0.01; ***P < 0.001; ****P < 0.0001

mTORC1 signaling is aberrantly activated in many cancers, including LSCC [25]. To examine whether the newly discovered mTORC1 regulation of the ERO1α/SLC7A11 pathway is also detectable in human cancer cells, a mTORC1-hyperactivated LSCC cell line, LIU-LSC-1, was employed [25]. As shown in Fig. 7A and B, the knockdown of Raptor led to the downregulation of ERO1α, IL-6, p-STAT3, and SLC7A11 in LIU-LSC-1 cells. Inhibition of mTORC1 with rapamycin achieved a similar result (Supplementary Fig. 8A). Moreover, manipulation of the activity of mTORC1 by TSC2 knockout increased the expressions of ERO1α, IL-6, p-STAT3, and SLC7A11 in TU177 cells (an LSCC cell line with moderate mTORC1 activity) (Supplementary Fig. 8B). In addition, genetic or pharmacological inhibition of mTORC1 led to a similar result in other types of mTORC1-activated cancer cells, such as PANC-1, MDA-MB-231, A549 and HeLa (Supplementary Fig. 8C, D). Because mTORC1 regulates the ERO1α/SLC7A11 signaling network in multiple human cancer cell lines in vitro, we predicted that this signaling regulation should also exist in human tumors in vivo. As depicted in Fig. 7C and D and Supplementary Fig. 8E, ERO1α, IL-6, p-STAT3, and SLC7A11 levels were substantially elevated in most of the 24 LSCC tissues tested, and p-S6 levels were significantly enhanced in these tissues, but not in the corresponding ANM tissues. Therefore, the regulatory pathway of SLC7A11 by the ERO1α/IL-6/STAT3 axis presents in human cancer cells with hyperactivated mTORC1 signaling.

To confirm the pro-oncogenic and anti-ferroptotic roles of ERO1α in human cancer cells, two shRNAs, especially targeting ERO1α were transfected into LIU-LSC-1 cells. The knockdown efficiency was confirmed by western blotting (Fig. 7E). Consistent with MEFs, the depletion of ERO1α led to downregulated expression of IL-6, p-STAT3, and SLC7A11 in LIU-LSC-1 cells (Fig. 7E). We also confirmed that knockdown of ERO1α reduced the proliferative and angiogenic capacities of LIU-LSC-1 cells (Fig. 7F–I). Not surprisingly, in line with MEFs, LIU-LSC-1 shERO1α cells exhibited a greater susceptibility to erastin, which could be rescued by Lip-1 (Fig. 7J, K). Notably, ERO1α knockdown cells exhibited increased lipid peroxidation (L-ROS and MDA) and GSH depletion (Fig. 7L–N). Additionally, through the subcutaneous injection of LIU-LSC-1 cells in three groups (shSc + vehicle, shERO1α^1^ + vehicle, and shERO1α^1^ + Lip-1) of nude mice, xenograft animal models were obtained to confirm the anti-ferroptosis effects of ERO1α again. Repression of ERO1α reduced the sizes of developed tumors and increased MDA levels, which did not occur in the control group. However, Lip-1 partially reverses the growth inhibition and ferroptotic events induced by silencing ERO1α (Fig. 7O–R). Overall, the mTORC1/ERO1α/IL-6/STAT3/SLC7A11 signaling cascade, which is also present in human cancer cells, plays a critical role in ferroptosis resistance and tumor progression.

### Targeting ERO1α significantly enhances the antitumor effect of IKE

The data above revealed that ERO1α significantly contributed to tumor progression and ferroptosis resistance, encouraging us to explore whether inhibition of ERO1α in combination with ferroptosis inducers could synergistically inhibit the growth of mTORC1-activated cells in vivo. sgERO1α NTC/T1 null cells and control cells were inoculated into nude mice to form subcutaneous tumors. The mice were treated with IKE, a metabolically stable analog of erastin, or vehicle [43] (Supplementary Fig. 9A). Consistent with our in vitro observations, ERO1α knockout NTC/T1 cells were more sensitive to IKE-induced tumor suppression than control cells in vivo (Supplementary Fig. 9B–D). IHC staining further revealed decreased Ki-67 and increased 4-HNE staining after IKE treatment, and these effects were markedly strengthened by ERO1α depletion (Supplementary Fig. 9E). Moreover, ERO1α deficiency resulted in therapy sensitivity, which was associated with increased MDA levels (Supplementary Fig. 9F). Notably, there was no loss of body weight in the treated mice (Supplementary Fig. 9G).

**Fig. 8:**
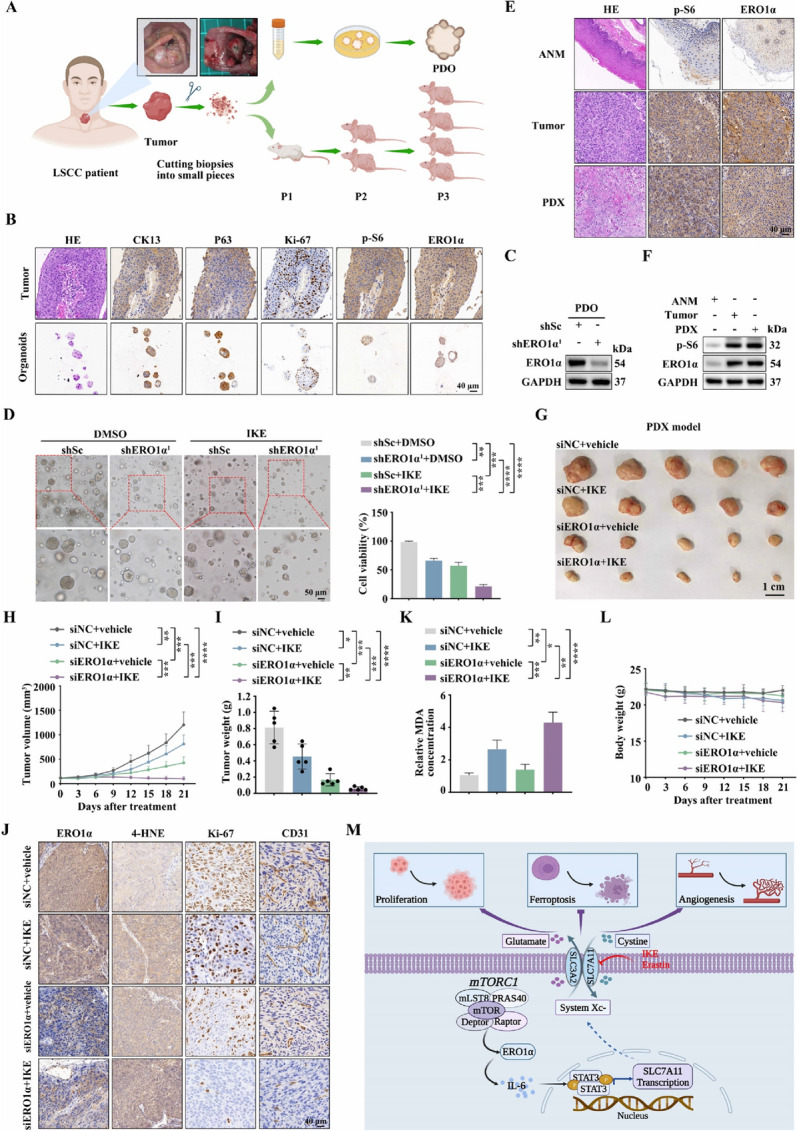
The combination of ERO1α inhibition and IKE exerts an effective inhibitory effect on the growth of PDO and PDX models. **A** Schematic workflow of the generation of LSCC organoids and PDX models. **B** Representative IHC images of CK13, p63, Ki-67, p-S6, and ERO1α staining from LSCC tissues and organoids. Scale bar, 40 μm. **C** LSCC organoids were infected with lentiviruses expressing shRNAs targeting ERO1α (shERO1α^1^) or a non-targeting shRNA (shSc). The level of ERO1α was detected by western blotting. **D** shERO1α.^1^ or shSc lentiviruses-infected organoids were treated with IKE (50 μM) or DMSO for 24 h. The cell viability of organoids was determined by Cell-Titer Glo-3D cell viability assay. Left panels: representative phase contrast images. Right panels: quantitation of the data. Scale bar, 50 μm. **E** and **F** The expression of p-S6 and ERO1α in the PDX tumor tissues, primary tumor tissues and ANM tissues were analyzed by IHC (**E**) and western blotting (**F**). **G**–**I** Tumor images (**G**), tumor volume (**H**), and tumor weight (**I**) of PDX model tumors treated with ERO1α siRNAs or siNC, together with IKE (40 mg/kg) or vehicle. *n* = 5 mice per group. **J** IHC staining of PDX tumor tissues using the indicated antibodies. Scale bar, 40 μm. **K** MDA assay was used to detected lipid peroxidation levels in randomly selected PDX tumor section. **L** Body weight of mice. **M** Schematic illustration of the activated ERO1α/IL-6/STAT3/SLC7A11 pathway is critical for mTORC1-mediated ferroptosis resistance and tumor progression. Error bars indicate mean ± SD of triplicate (if mentioned otherwise) samples. *P < 0.05; **P < 0.01; ***P < 0.001; ****P < 0.0001

Considering that PDO and PDX tumor models can highly preserve the heterogeneity and histological characteristics of the original tumors [44], we further tested the therapeutic potential of combining ERO1α inhibition and IKE in tumor treatment using PDO and PDX models (Fig. 8A). As shown in Fig. 8B, histological analysis confirmed that these LSCC organoids retained the histological features of the original tumors. Similar to our observations in the CDX models, the depletion of ERO1α significantly aggravated the inhibitory effect of IKE on the growth of LSCC organoids (Fig. 8C, D). Furthermore, fresh LSCC tumors with activated mTORC1 and high levels of ERO1α were chosen to establish the PDX models (Fig. 8E, F). Because no current ERO1α inhibitor is suitable for in vivo treatment, we used ERO1α siRNAs to knock down ERO1α in our animal studies. We showed that co-treatment with ERO1α siRNAs and IKE suppressed PDX tumor growth much more potently than either treatment alone (Fig. 8G–I). Successful in vivo knockdown of ERO1α in tumors by siRNAs was confirmed by IHC analysis (Fig. 8J). Further analyses revealed that the combination treatment, which was well tolerated in vivo, synergistically increased the staining of 4-HNE and the production of MDA in PDX tumor samples (Fig. 8J–L). Our results show that ERO1α inhibition sensitizes mTORC1-activated cells to ferroptosis. This suggests combining ERO1α inhibition with ferroptosis inducers in the treatment of mTORC1-related cancer to achieve better outcomes.

## Discussion

Increasing research indicates that mTORC1, which is aberrantly activated in many types of human cancers, is critical for promoting tumor development [6, 8]. However, its underlying mechanisms still need to be fully understood. Currently, even though rapamycin and its derivatives have been approved for use in the treatment of several mTORC1-associated tumors, such as breast cancer, advanced renal cancer, and tuberous sclerosis complex, their clinical therapeutic effects are minimal [45–47]. One key reason for this is that suppression of mTORC1 leads to feedback activation of AKT [46]. Therefore, it is urgent to identify novel therapeutic targets and develop new approaches for mTORC1-related cancers. In this study, which primarily focused on mTORC1-activated MEFs and cancer cell lines, LSCC tissues, organoids, and PDX models, we identified ERO1α as a functional downstream target of mTORC1. We also illustrated that elevated ERO1α expression contributes to tumor progression driven by mTORC1 activation. In addition, we found that mTORC1 promotes the transcription of ERO1α by upregulating the transcription factor HIF-1α, a well-known mTORC1 downstream effector. This agree with previous findings suggesting that ERO1α is a hypoxia-inducible gene in many cell lines [48]. Recently, the crucial role of ERO1α in tumor progression and treatment has been illustrated in many types of human cancers [49–54]. For example, Zhang and colleagues reported that ERO1α was significantly overexpressed and promoted cell proliferation and tumor growth in pancreatic ductal adenocarcinoma (PDAC) [49]. Tanaka et al. illustrated that ERO1α levels were related to the number of blood vessels in triple-negative breast cancer (TNBC) samples. Moreover, the expression of ERO1α promoted tumor growth by augmenting angiogenesis [53]. Our findings correlate with the results of previous studies, suggesting that ERO1α is critical for cell proliferation and angiogenesis and unveiling a new molecular link between mTORC1 activation and tumor development. Because aberrations in the PI3K/AKT/mTORC1 pathway are common in PDAC and TNBC [55, 56], it is likely that hyperactivated mTORC1 in these cancers upregulates ERO1α expression and then promotes tumor progression. In line with this, inhibition of mTORC1 led to a significantly downregulated expression of ERO1α in the PDAC cells PANC-1 and the breast cancer cells MDA-MB-231 (Supplementary Fig. 8C, D). Therefore, ERO1α is a potential therapeutic target for mTORC1-related cancers.

Inducing ferroptosis in tumor cells has promising potential for cancer treatment, but its regulatory networks remain largely unclear. ERO1α is an endoplasmic reticulum (ER) stress–related gene, which improves cell perseverance against challenges of high levels of protein misfolding during ER stress by retaining the good activity of oxidative protein folding [49, 50, 57]. In this study, in addition to accelerating cell proliferation and promoting angiogenesis, we found that ERO1α plays a critical role in ferroptosis resistance driven by mTORC1 based on the following evidence. First, knockout or knockdown of ERO1α accelerated erastin-induced lipid peroxidation and ferroptic death in mTORC1-activated cells, while overexpression of ERO1α attenuated ferroptosis triggered by erastin in control cells. Second, the anti-ferroptosis role of ERO1α was further confirmed by the observation that inhibition of ferroptosis using Lip-1 partly rescued ERO1α depletion–mediated suppression of tumor growth and upregulation of MDA. Therefore, in addition to relieving ER stress caused by the massive synthesis of new proteins due to mTORC1 activation [58], elevated ERO1α can protect mTORC1-activated cells against ferroptosis. Furthermore, through integration analysis of RNA-seq data and subsequent functional validation, we found that elevated ERO1α promotes ferroptosis resistance through upregulation of SLC7A11 in response to mTORC1 activation. This discovery confirmed the previous findings that SLC7A11 is critical for mTORC1-mediated glutathione synthesis [59] and unveiled a novel upstream regulator of SLC7A11. Thus, it is vital to sensitize mTORC1-activated cells to ferroptosis inducers by targeting this newly discovered ERO1α/SLC7A11 pathway. In line with this, we showed that targeting ERO1α, combined with the ferroptosis inducer IKE, has an excellent antitumor effect in three mTORC1-activated models, including NTC/T1 null cell xenografts, LSCC organoids, and LSCC PDX models. Therefore, ERO1α inhibition combined with ferroptosis induction constitutes a new and effective therapeutic strategy for some mTORC1-related cancers.

Angiogenesis, one of the hallmarks of cancer, facilitates tumor growth by playing a critical role in the delivery of oxygen and nutrients [60]. Previous studies have demonstrated that ERO1α is involved in tumor angiogenesis. To date, the angiogenesis-promoting effects of ERO1α have been linked to increased expression and secretion of vascular endothelial growth factor [51, 53, 61, 62]. In addition to ferroptosis resistance and cell proliferation, we propose here that ERO1α also at least partially promotes angiogenesis via upregulation of SLC7A11 in mTORC1-activated cells. To our knowledge, this is the first report of direct evidence supporting SLC7A11 with the function of promoting angiogenesis. In addition, these findings provide a mechanistic explanation for a previous observation that administration of the SLC7A11 inhibitor erastin diminished angiogenesis in glioma [63]. However, it has not been investigated whether the angiogenesis-promoting effect of SLC7A11 depends on or is independent of its downstream GSH/GPX4 pathway. Future studies need to explore this issue.

Because SLC7A11 is frequently overexpressed in human malignancies and plays a significant role in ferroptosis resistance and tumor progression [64], it is critical to explore how SLC7A11 expression is controlled. An increasing number of studies suggest that the expression of SLC7A11 is finely regulated at the transcription level by transcription factors, at the post-transcription level by RNA binding proteins or noncoding RNAs, and at the protein degradation level by proteasome [59, 65–73]. In this study, we illustrated that ERO1α stimulates STAT3 activity through upregulation of IL-6. Subsequently, activated STAT3 promotes the transcription of SLC7A11 by directly binding with the promoter of the *SLC7A11* gene. We identified IL-6 as a novel target of ERO1α and confirmed the suggestion by previous studies conducted using other cancer cell models that STAT3 directly transcriptional regulates SLC7A11 [74, 75]. In addition, this may help to explain why IL-6 is elevated in mTORC1-related tumors. For example, IL-6 is significantly upregulated and plays a critical role in the development of tuberous sclerosis complex, a benign tumor syndrome caused by aberrant mTORC1 activation due to the loss of either TSC1 or TSC2 [76]. Considering our findings, overactivated mTORC1 likely facilitates the expression of IL-6 through upregulation of ERO1α, thus promoting cell proliferation and tumor growth in the TSC. Interestingly, it has also been reported that STAT3 upregulates SLC7A11 through p53 downregulation, thus alleviating p53-mediated transcriptional inhibition of SLC7A11 [77]. This scenario could be ruled out because the Tsc2 − / − MEFs used here were p53 null. Furthermore, a limitation of this study is that the cytokine antibody array kit we used only covers 40 common cytokines. Investigating whether other cytokines are also involved in ERO1α-mediated upregulation of SLC7A11 in the future would be interesting. Furthermore, it is worth exploring whether ERO1α directly affects the formation of disulfide bonds in SLC7A11, thereby affecting its expression level and activity.

In summary, we have demonstrated that aberrantly activated mTORC1 contributes to ferroptosis resistance and tumor growth by regulating of the ERO1α/IL-6/STAT3/SLC7A11 signaling pathway (Fig. 8M). Our findings will help in the elucidation of the molecular mechanism by which dysregulated mTORC1 signaling drives resistance to ferroptosis and tumorigenesis, indicating that combining ERO1α inhibition with ferroptosis inducers may be a novel therapeutic strategy for the treatment of mTORC1-related tumors.

### Supplementary Information

**Supplementary Material 1.**

**Supplementary Material 2. **

**Supplementary Material 3.**

**Supplementary Material 4.**

**Supplementary Material 5. **

## Data Availability

All data generated and analyzed during this study are included in this published article and supplementary information. Materials generated during the present study are available from the corresponding author on reasonable request. The GEO accession number for RNA-seq data is GSE246899.

## Abbreviations

mTOR: Mechanistic target of rapamycin
mTORC1: Mammalian target of rapamycin complex 1
mTORC2: Mechanistic target of rapamycin complex 2
TSC1: Tuberous sclerosis 1
TSC2: Tuberous sclerosis 2
Rheb: Ras homologue enriched in brain
AKT: Protein kinase B
GSH: Glutathione
L-ROS: Lipid reactive oxygen species
MDA: Malondialdehyde
GPX4: Glutathione peroxidase 4
SLC7A11: Solute carrier family 7 member 11
Lip-1: Liproxstatin-1
MEFs: Mouse embryonic fibroblasts
LSCC: Laryngeal squamous cell carcinoma
PDO: Patient-derived Oragnoid
PDX: Patient-derived xenograft
ERO1α: Endoplasmic reticulum oxidoreductase 1 alpha
IL-6: Interleukin-6
STAT3: Signal transducer and activator of transcription 3
RSL3: RAS-selective lethal 3
IKE: Imidazole ketone erastin
DFX: Deferoxamine
MIG: Monokine induced by interferon-gamma
MIP-1α: Macrophage inflammatory protein-1alpha
HUVECs: Human umbilical vein endothelial cells
HEK 293T: Human embryonic kidney 293T
ANM: Adjacent normal tissues
qRT**–**PCR: Quantitative real-time PCR
IF: Immunofluorescence
TEM: Transmission electron microscopy
ChIP: Chromatin immunoprecipitation
CCK-8: Cell Counting Kit-8
CAM: Chicken chorioallantoic membrane
ELISA: Enzyme-linked immunosorbent assay
IHC: Immunohistochemistry
ELT3: Tsc2-null rat uterine leiomyoma cells
hiPSCs: Human induced pluripotent stem cells
HK2: Hexokinase
ENO3: Enolase 3
siRNAs: Small interfering RNA
HIF-1α: Hypoxia-inducible factor-1
shRNA: Short hairpin RNA
DEGs: Differentially expressed genes
4-HNE: 4-Hydroxynonenal
PDAC: Pancreatic ductal adenocarcinoma
TNBC: Triple-negative breast cancer
