# Supplementary material for: Augmented ERO1α upon mTORC1 activation induces ferroptosis resistance and tumor progression via upregulation of SLC7A11
Source: J Exp Clin Cancer Res. 2024 Apr 13;43:112. doi: 10.1186/s13046-024-03039-2 (PMC11015652; doi:10.1186/s13046-024-03039-2)
Supplement: Supplementary file 1 — Supplementary Material 1. [file 13046_2024_3039_MOESM1_ESM.docx]

**Supplementary Table S1. Antibodies used in this study.**

| **Antibody** | **Source** | **Application** |
| --- | --- | --- |
| Tsc1 | Cell Signaling Technology (Cat#6935S) | 1:1000 for WB |
| Tsc2 | Cell Signaling Technology (Cat#4308S) | 1:1000 for WB |
| ERO1α | Proteintech (Cat#67416-1-Ig) | 1:5000 for WB; 1:500 for IHC; 1:200 for IF |
| p-P70S6K | Abcam (Cat#ab2571) | 1:1000 for WB |
| P70S6K | Abcam (Cat#ab32529) | 1:5000 for WB |
| p-S6 (Ser235/236) | Cell Signaling Technology (Cat#4858S) | 1:2000 for WB; 1:200 for IHC |
| S6 | Cell Signaling Technology (Cat#2217S) | 1:1000 for WB |
| β-actin | Sigma-Aldrich (Cat#A1978) | 1:1000 for WB |
| mTOR | Cell Signaling Technology (Cat#2983) | 1:1000 for WB |
| Raptor | Cell Signaling Technology (Cat#2280S) | 1:1000 for WB |
| SLC7A11 | Abcam (Cat#ab307601) | 1:1000 for WB; 1:500 for IHC; 1:500 for IF |
| p-STAT3 (Tyr705) | Cell Signaling Technology (Cat#9145S) | 1:2000 for WB; 1:100 for IHC; 1:100 for ChIP |
| STAT3 | Cell Signaling Technology (Cat#9139S) | 1:1000 for WB; 1:800 for IF |
| IL-6 | ZEN BIO (Cat#500286) | 1:1000 for WB; 1:100 for IHC |
| GAPDH | Cell Signaling Technology (Cat#5174S) | 1:1000 for WB |
| Rictor | Cell Signaling Technology (Cat#2114S) | 1:1000 for WB |
| HIF-1α | Abcam (Cat#ab228649) | 1:1000 for WB |
| Ki-67 | Cell Signaling Technology (Cat#34330) | 1:200 for IHC |
| CD31 | Abcam (Cat#ab182981) | 1:2000 for IHC |
| P63 | Cell Signaling Technology (Cat#39692S) | 1:500 for IHC |
| CK13 | Proteintech (Cat#10164-2-AP) | 1:100 for IHC |
| 4-HNE | Abcam (Cat#ab48506) | 1:200 for IHC |

**Supplementary Table S2. Clinical characteristics of 24 LSCC patients.**

| **Gender** | **Age** | **TNM Stage** |
| --- | --- | --- |
| Male | 67 | T1 N0 M0 |
| Male | 67 | T4 NI M0 |
| Female | 52 | T1 N0 M0 |
| Male | 79 | T3 N1 M0 |
| Male | 77 | T3 N0 M0 |
| Male | 48 | T2 N0 M0 |
| Male | 48 | T4 N0 M0 |
| Female | 54 | T3 N0 M0 |
| Male | 56 | T1 N0 M0 |
| Male | 65 | T1 N0 M0 |
| Male | 75 | T3 N0 M0 |
| Male | 68 | T1 N0 M0 |
| Male | 53 | T1 N0 M0 |
| Male | 67 | T3 N0 M0 |
| Male | 59 | T1 N0 M0 |
| Male | 78 | T3 N0 M0 |
| Male | 73 | T2 N0 M0 |
| Male | 64 | T4 N2 M0 |
| Male | 70 | T4 N0 M0 |
| Male | 52 | T1 N0 M0 |
| Male | 78 | T1 N0 M0 |
| Male | 59 | T3 N0 M0 |
| Male | 77 | T2 N0 M0 |
| Male | 51 | T1 N0 M0 |

**Supplementary Table S3. Targeting sequences for shRNẠs used in this study.**

| **Note** | **Sequences (5'-3')** |
| --- | --- |
| (mus)/(human) shSc | TTCTCCGAACGTGTCACGT |
| (mus) shERO1α^1^ | GGCTACACTTTAGGAACATTT |
| (mus) shERO1α^2^ | AGCTAACCAGACAAGAAATAG |
| (mus) shSLC7A11^1^ | CTGTGGTGATGGTCCTAAATA |
| (mus) shSLC7A11^2^ | TTCGCTGTCTCCAGGTTATTC |
| (human) shERO1α^1^ | GAGCATTCTACAGACTTATAT |
| (human) shERO1α^2^ | GGATGATTGTACCTGTGATGT |

**Supplementary Table S4. Primer sequences for qRT-PCR used in this study.**

| **Primer name** | **Sequence (5' to 3')** | **Source** |
| --- | --- | --- |
| (mus) ERO1α forward | GTTAGTGGTTACCTGGACGACT | Sangon Biotech  (Shanghai, China) |
| (mus) ERO1α  reverse | CAGAATACTTGTAGCTCGCAGAC | Sangon Biotech  (Shanghai, China) |
| (mus) SLC7A11 forward | AAGTCTAATGGGGTTGCCCT | Sangon Biotech  (Shanghai, China) |
| (mus) SLC7A11 reverse | TGATAGCCATGGAGATGCAG | Sangon Biotech  (Shanghai, China) |
| (mus) β-actin  forward | AGAGGGAAATCGTGCGTGAC | Sangon Biotech  (Shanghai, China) |
| (mus) β-actin  reverse | CAATAGTGATGACCTGGCCGT | Sangon Biotech  (Shanghai, China) |
| (human) ERO1α forward | GGCTGGGGATTCTTGTTTGG | Sangon Biotech  (Shanghai, China) |
| (human) ERO1α reverse | AGTAACCACTAACCTGGCAGA | Sangon Biotech  (Shanghai, China) |
| (human) SLC7A11 forward | GGTGGTGTGTTTGCTGTC | Sangon Biotech  (Shanghai, China) |
| (human) SLC7A11 reverse | GCTGGTAGAGGAGTGTGC | Sangon Biotech  (Shanghai, China) |
| (human) β-actin forward | ATCGTCCACCGCAAATGCTTCTA | Sangon Biotech  (Shanghai, China) |
| (human) β-actin reverse | AGCCATGCCAATCTCATCTTGTT | Sangon Biotech  (Shanghai, China) |

**Supplementary Table S5. Targeting sequences for siRNAs used in this study.**

| **Note** | **Sequences (5'-3')** |
| --- | --- |
| (mus)/(human) Negative control | TTCTCCGAACGTGTCACGT |
| (mouse) mTOR^1^ | GAACTCGCTGATCCAGATG |
| (mouse) mTOR^2^ | GGGTCATGAACACGTCAAT |
| (mouse) Raptor^1^ | GGACAACGGTCACAAGTAC |
| (mouse) Raptor^2^ | GGCTAGTCTGTTTCGAAAT |
| (mouse) Rictor | GCGAGCTGATGTAGAATTG |
| (mouse) IL-6^1^ | AGTCGGAGGCTTAATTACA |
| (mouse) IL-6^2^ | CAGGAAATTTGCCTATTGA |
| (mouse) STAT3^1^ | CTGGATAACTTCATTAGCA |
| (mouse) STAT3^2^ | GGGTACATCATGGGTTTCA |
| (mouse) HIF-1α^1^ | GCCTAACAGTCCCAGTGAA |
| (mouse) HIF-1α^2^ | GCTGACCAGTTACGATTGT |
| (human) ERO1α | GAGCATTCTACAGACTTAT |
| (human) Raptor | GGACAACGGCCACAAGUAC |

.

**Supplementary Table S6. Primers used for luciferase reporter in this study.**

|  | **Gene** | **Forward primers (5'-3')** | **Reverse primers (5'-3')** |
| --- | --- | --- | --- |
| Wild-type | SLC7A11 | TGTGTCTGTTCATGCGG  AGCTGTAT | CCTTCGCTGGCTCTATAATTACTCC |
| Mutated | Site 1 | AGTGTTGCTACCGTACA  CCCGGGG | GTCAAATCTAATATTTACAAAGTC |
| Mutated | Site 2 | CTTTGTTCTGCCTGATGCTTTGGATCC | CATACTCTTAAAGAGGGG |

| **Supplementary Table S7. Primers used for ChIP Assays in this study.** | | |
| --- | --- | --- |
|  | **Sense (5'-3')** | **Antisense (5'-3')** |
| Site 1 (PCR) | ATTCCCTGGTTCCCTG  TTTACT | AATTCTCCAGGTTTGCATCAG |
| Site 2 (PCR) | TGTCTGTTCATGCGGA  GCTGTA | CCTTAGGGCTGCTCTGTTTG |
| NBR (qRT-PCR) | GCGCCCTGTCTATGCT  CATTCA | CTGCCCGCCCTGTTC  CATCT |
| PBR (qRT-PCR) | CACGTGAGTAACTGGTCTGGGATAGG | CCGGTCCTTGGTCCATTCCC |
