# Supplementary material for: Augmented ERO1α upon mTORC1 activation induces ferroptosis resistance and tumor progression via upregulation of SLC7A11
Source: J Exp Clin Cancer Res. 2024 Apr 13;43:112. doi: 10.1186/s13046-024-03039-2 (PMC11015652; doi:10.1186/s13046-024-03039-2)
Supplement: Supplementary file 4 — Supplementary Material 4. [file 13046_2024_3039_MOESM4_ESM.docx]

**_­_Supplementary Figures and Legends**

**
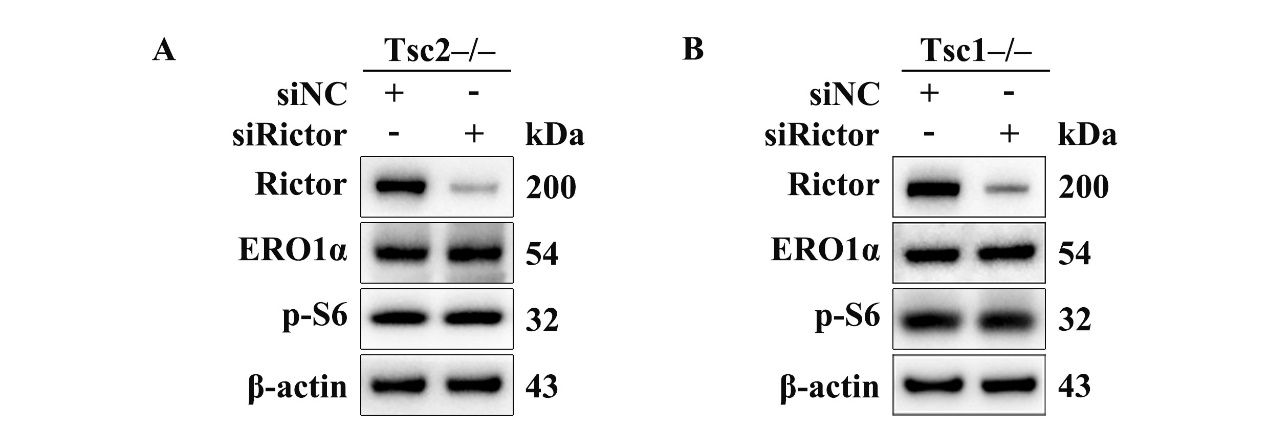
**

**Supplementary Fig. 1: Inhibition of mTORC2 has little effect on the expression of ERO1α.**

Tsc2−/− (A) or Tsc1−/− (B) MEFs were transfected with siRNA targeting Rictor (siRictor) or the control (siNC) for 48 hours. Cell lysates were collected for western blotting with the indicated antibodies.

**
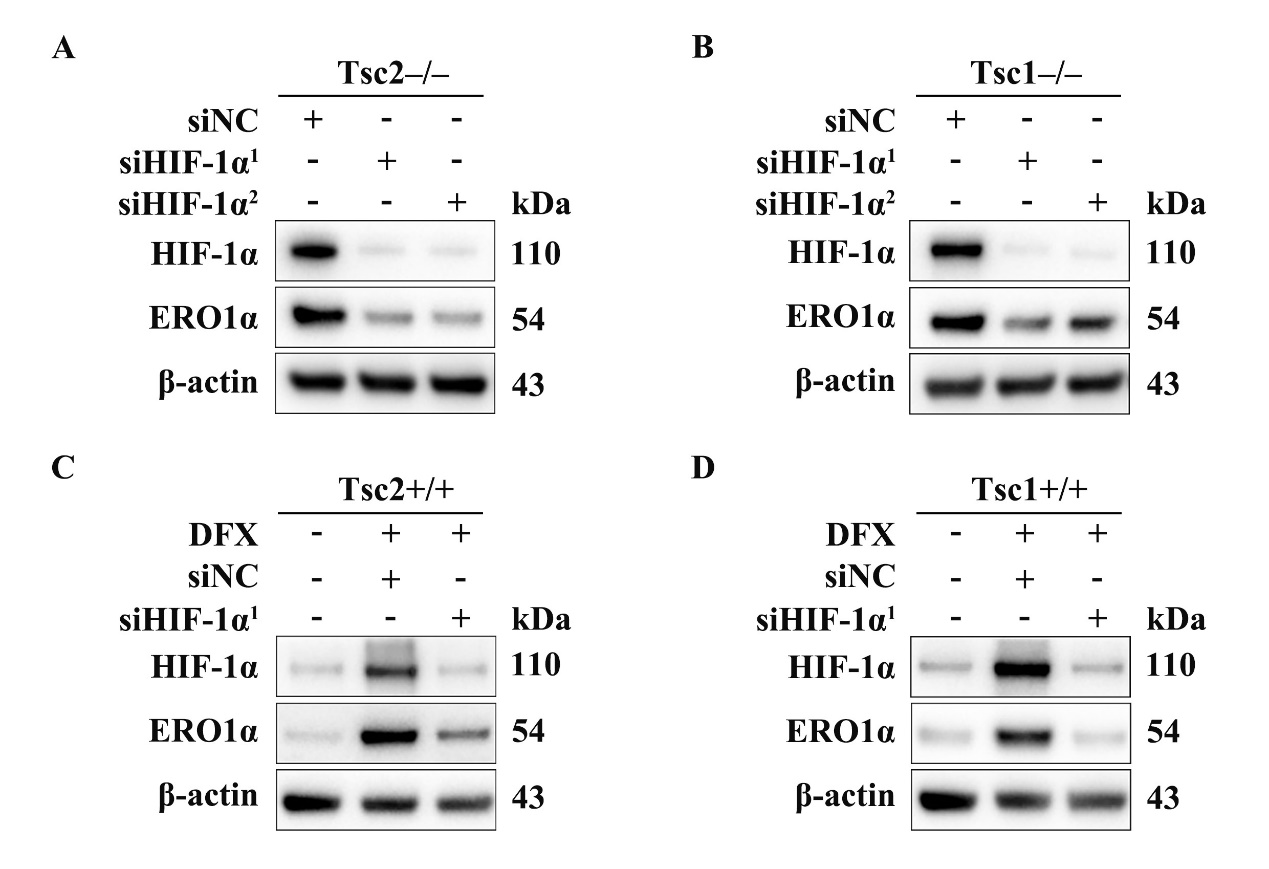
**

**Supplementary Fig. 2: mTORC1 upregulates ERO1α through HIF-1α.**

(A and B) Tsc2−/− (A) and Tsc1−/− (B) MEFs were transfected with siRNA targeting HIF-1α or the control (siNC) for 48 hours. (C and D) After transfected with siRNA targeting HIF-1α or the control (siNC) for 48 hours, Tsc2+/+ (C) and Tsc1+/+ (D) MEFs were treated with 200 μM DFX for 24 hours. (A–D) Cell lysates were collected for western blotting using the indicated antibodies.

**
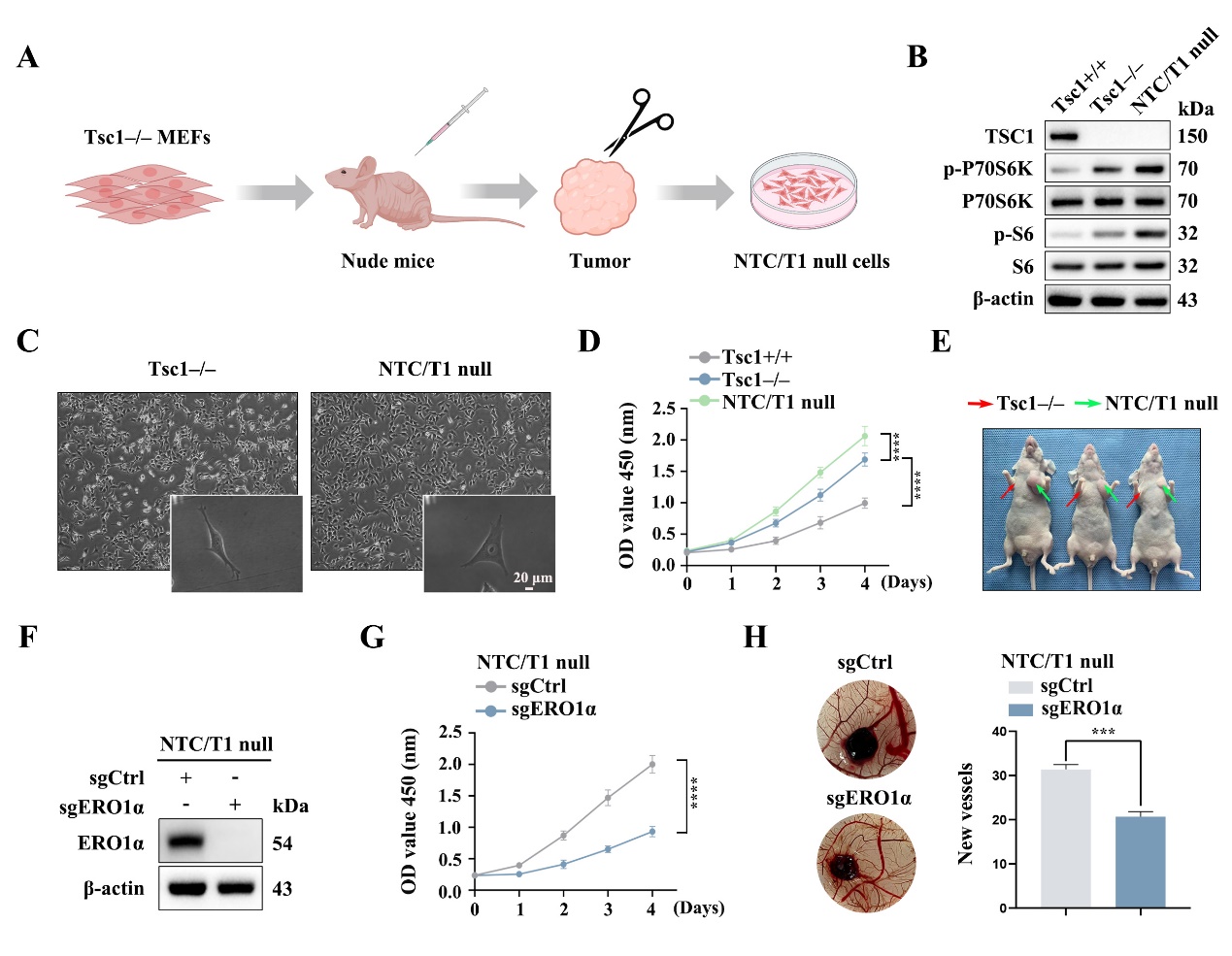
**

**Supplementary Fig. 3:** **Establishment and characterization of a novel cell line, NTC/T1 null.**

(A) Flowchart of NTC/T1 null cell line establishment based on Tsc1−/− MEFs-derived xenografts. (B) The indicated cells were subjected to immunoblotting with TSC1, p-P70S6K and p-S6 antibodies. (C) Representative images of Tsc1−/− MEFs and NTC/T1 null cells. Scale bar, 20 μm. (D) CCK-8 assay was performed to evaluate cell growth rates of the indicated cells. (E) Tsc1−/− MEFs and NTC/T1 null cells (3×10^6^) were subcutaneously inoculated into nude mice (N=3). After 30 days, the mice were anesthetized and photographed. (F–H) sgCtrl and sgERO1α NTC/T1 null cells were subjected to western blotting (F), CCK-8 (G), and CAM (H, left panels: representative images; right panels: quantifications) assays. Error bars indicate mean ± SD of triplicate samples. ****P*<0.001; *****P*<0.0001.

**
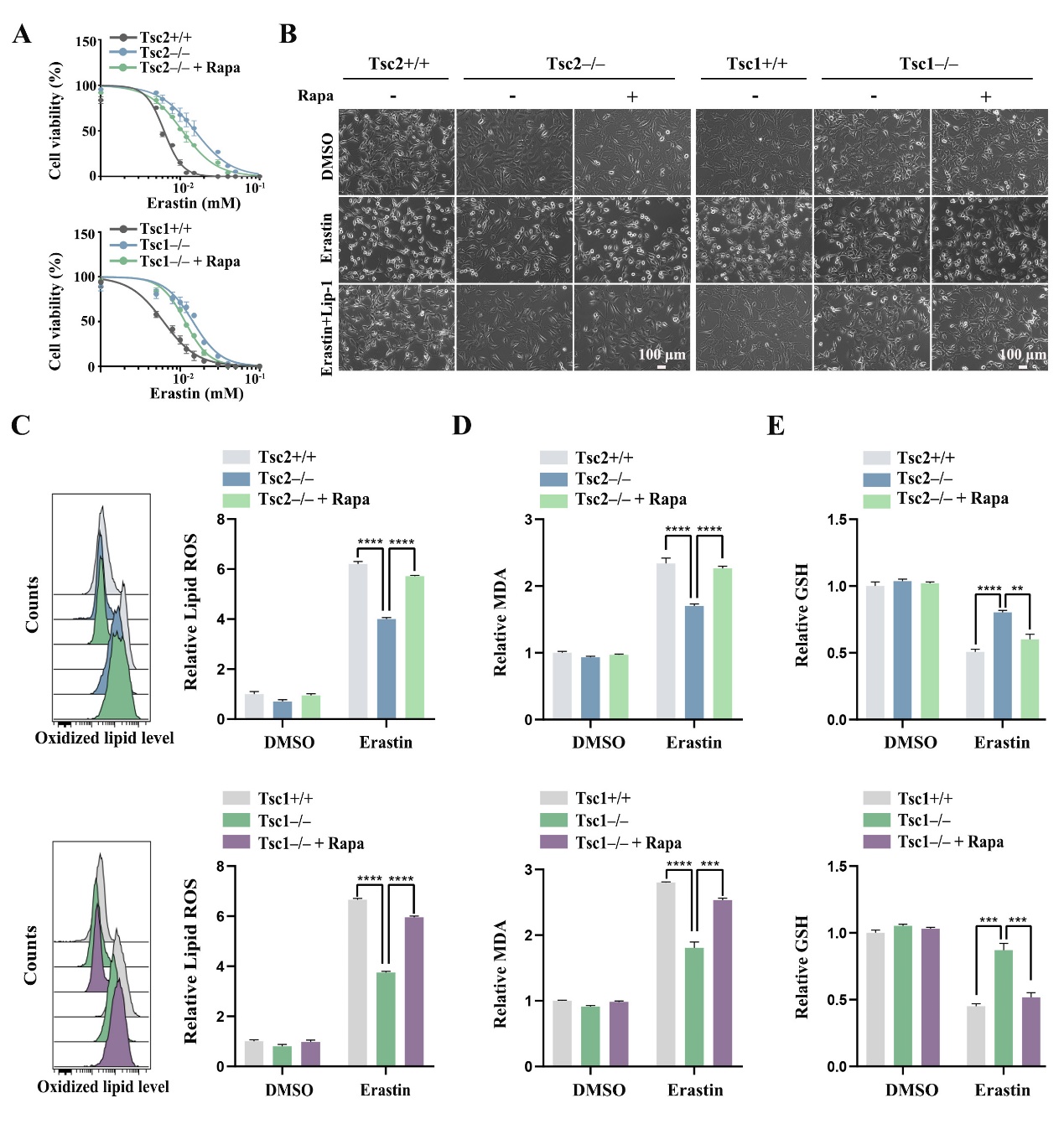
**

**Supplementary Fig. 4: mTORC1 promotes ferroptosis resistance.**

(A–E) Tsc2+/+, Tsc2−/−, rapamycin-pretreated (20 nM, 24 hours) Tsc2−/−, Tsc1+/+, Tsc1−/−, rapamycin-pretreated (20 nM, 24 hours) Tsc1−/− MEFs. (A) Cell viability of the indicated cells following treatment with erastin for 24 hours. (B) The indicated cells were treated with erastin (10 μM, 24 hours) in the absence or presence of Lip-1 (1 μM). The corresponding phase contrast images are shown. Scale bar, 100 μm. (C–E) The indicated cells were treated with or without erastin (10 μM) for 24 hours, and then L-ROS (C), intracellular MDA (D), and intracellular GSH (E) were assayed. Error bars indicate mean ± SD of triplicate samples. ***P*<0.01; ****P*<0.001; *****P*<0.0001.

**
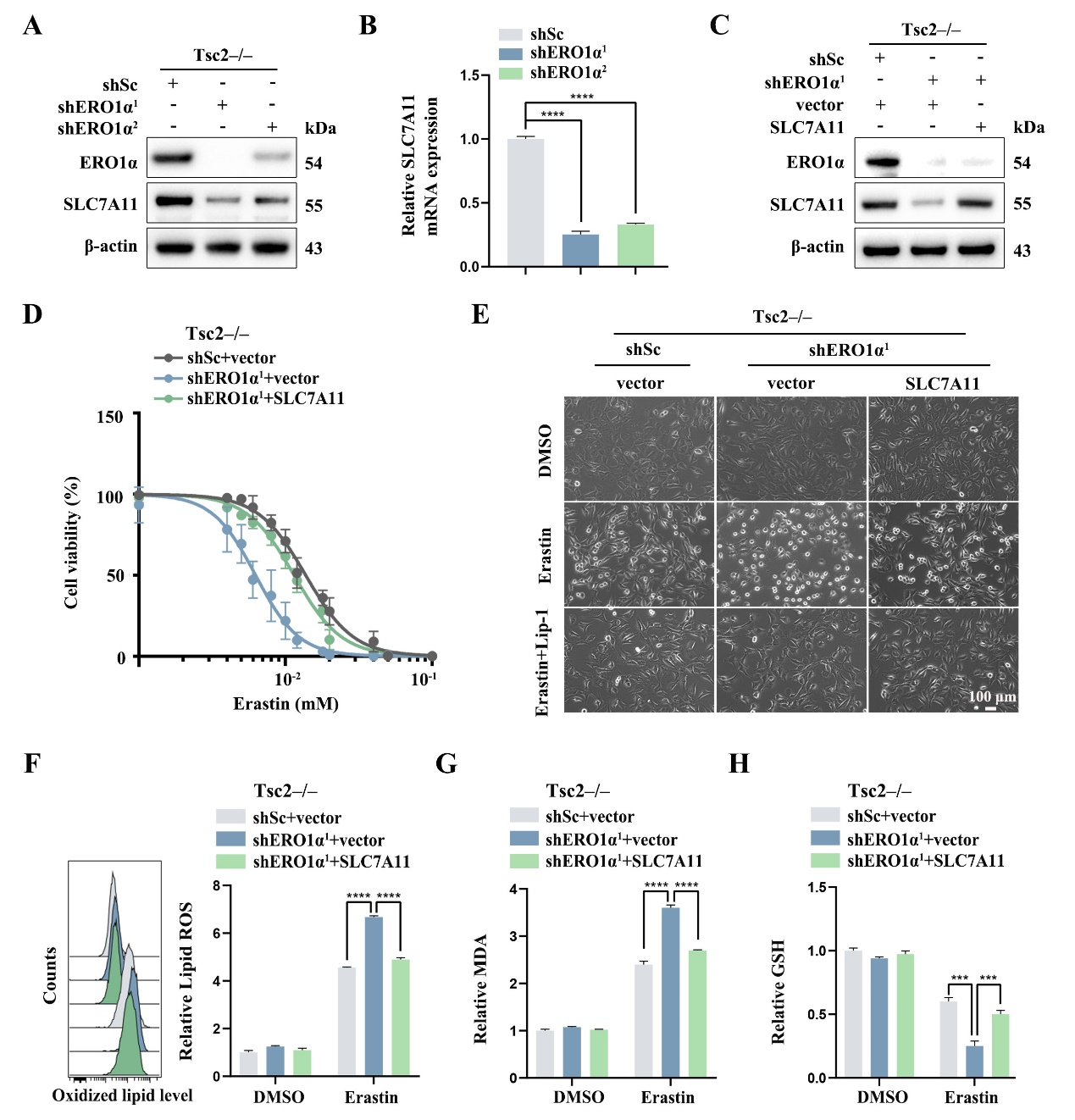
**

**Supplementary Fig. 5: Overexpression of SLC7A11 attenuates ERO1α depletion-induced ferroptosis in Tsc2−/− MEFs.**

(A and B) Tsc2−/− MEFs were transduced with ERO1α shRNAs-expressing (shERO1α^1^ or shERO1α^2^) lentiviruses or shSc. SLC7A11 expression was assessed by western blotting (A) and qRT-PCR (B). (C–H) shERO1α^1^ Tsc2−/− MEFs were infected with lentiviruses carrying an empty vector or expression vectors for SLC7A11. (C) Cell lysates were subjected to immunoblotting. (D) Cell viability was assessed after treatment with different concentrations of erastin for 24 hours in the indicated cells. (E) Representative phase-contrast images of the indicated cells treated with erastin (10 μM) in the absence or presence of Lip-1 (1 μM) for 24 hours. Scale bar, 100 μm. (F–H) The indicated cells were treated with or without erastin (10 μM) for 24 hours, and then L-ROS (F), intracellular MDA (G), and intracellular GSH (H) were measured. Error bars indicate mean ± SD of triplicate samples. ****P*<0.001; *****P*<0.0001.

**
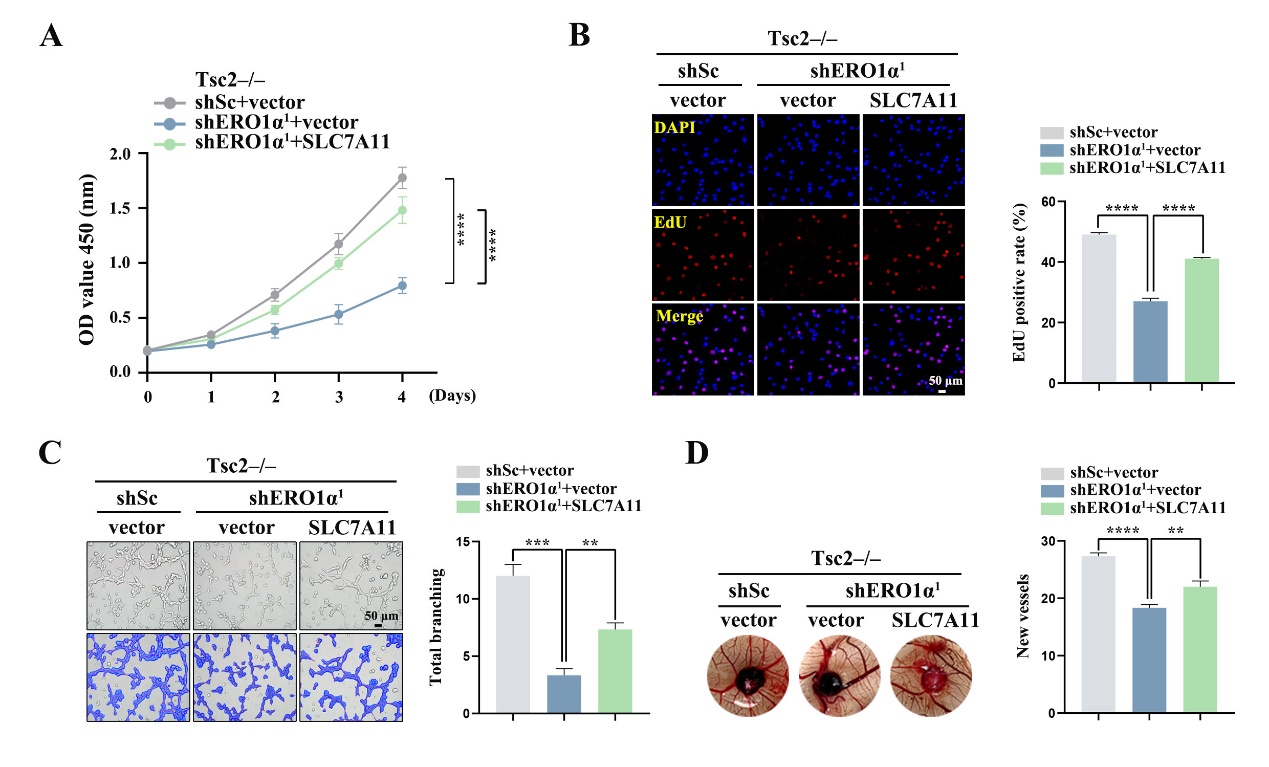
**

**Supplementary Fig. 6: Ectopic expression of SLC7A11 reverses the adverse effects of ERO1α knockdown on the proliferative and angiogenic abilities of Tsc2−/−** **MEFs.**

(A–D) shERO1α^1^ Tsc2−/− MEFs were infected with lentiviruses carrying an empty vector or expression vectors for SLC7A11. CCK-8 (A) and EdU (B) assays were performed to evaluate cell proliferation. Scale bar, 50 μm. The effect on angiogenesis was determined by tube formation (C) and CAM (D) assays. Representative images (left panels) and quantifications (right panels) were shown. Error bars indicate mean ± SD of triplicate samples. ***P*<0.01; ****P*<0.001; *****P*<0.0001.

**
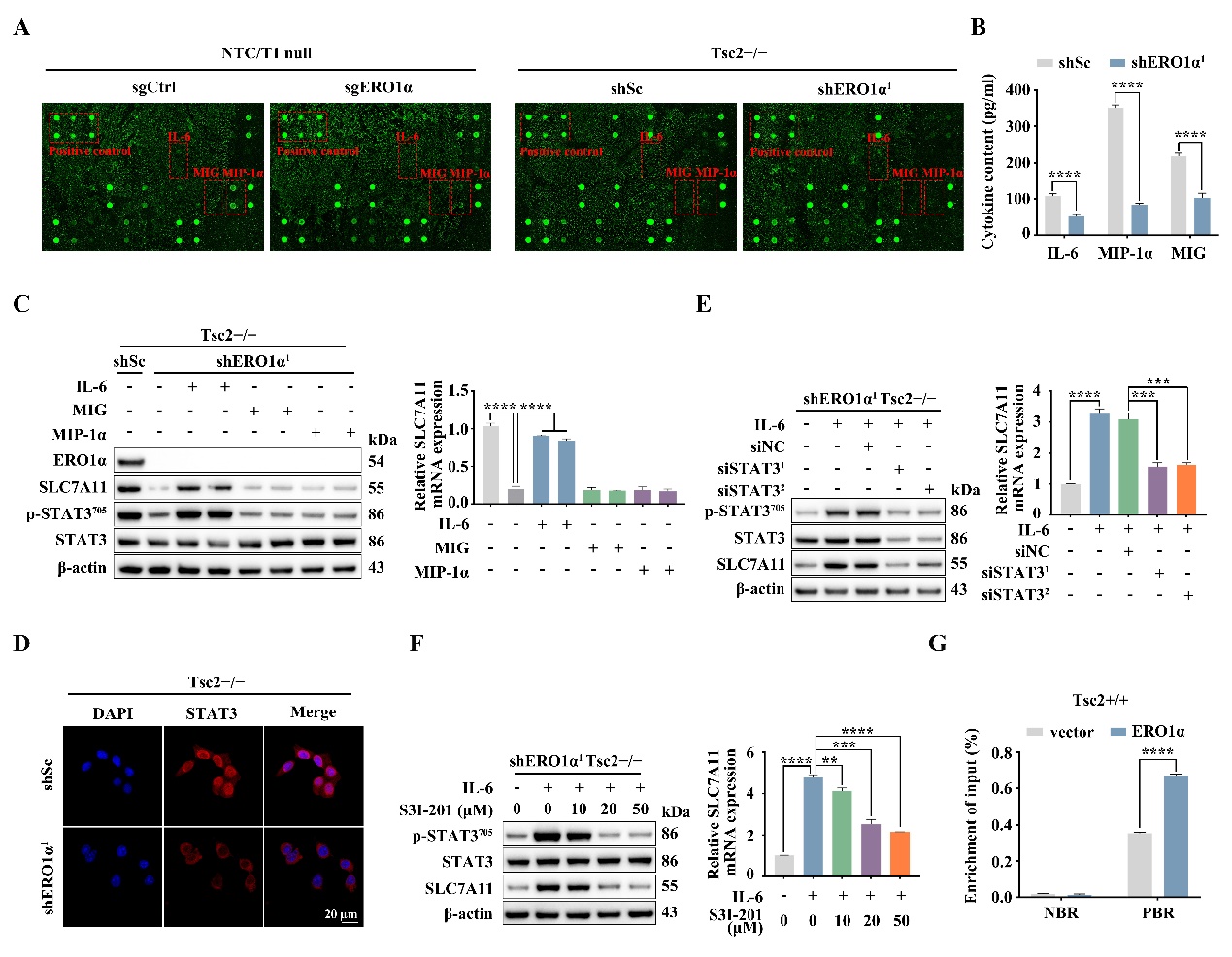
**

**Supplementary Fig. 7: ERO1α promotes SLC7A11 expression through activation of the IL-6/STAT3 pathway in Tsc2−/− MEFs.**

(A) sgERO1α NTC/T1 null cells, shERO1α^1^ Tsc2−/− MEFs and their corresponding control cells were subjected to cytokines array assays. (B) Cell supernatants from shERO1α^1^ Tsc2−/− MEFs and the control cells were collected, and IL-6, MIP-1α, and MIG levels were determined using an ELISA. (C) shERO1α^1^ Tsc2−/− MEFs were treated with IL-6 (20 ng/ml), MIP-1α (100 ng/ml), or MIG (100 ng/ml) for 24 hours. (D) Representative IF showing the cellular localization of STAT3 in the indicated cells. Scale bar, 20 μm. (E) IL-6 (20 ng/ml, 12 hours) pre-treated shERO1α^1^ Tsc2−/− MEFs were transfected with STAT3 siRNAs or control siRNA (siNC) for 48 hours. (F) IL-6 (20 ng/ml, 12 hours) pre-treated shERO1α^1^ Tsc2−/− MEFs were treated with S3I-201 for 24 hours. (C, E and F) Cell lysates were subjected to immunoblotting with the indicated antibodies (left panels), the mRNA level of SLC7A11 was detected by qRT-PCR (right panels). (G) ERO1α-overexpressing Tsc2+/+ MEFs and the control cells were subjected to ChIP analysis with antibodies to p-STAT3 or control rabbit IgG. qRT-PCR was performed to amplify regions surrounding the putative STAT3 binding Site 2 (PBR) and a nonspecific STAT3 binding region (NBR). The data were plotted as the ratio of immunoprecipitated DNA to total input DNA. Error bars indicate mean ± SD of triplicate samples. ***P*<0.01; ****P*<0.001; *****P*<0.0001.

**
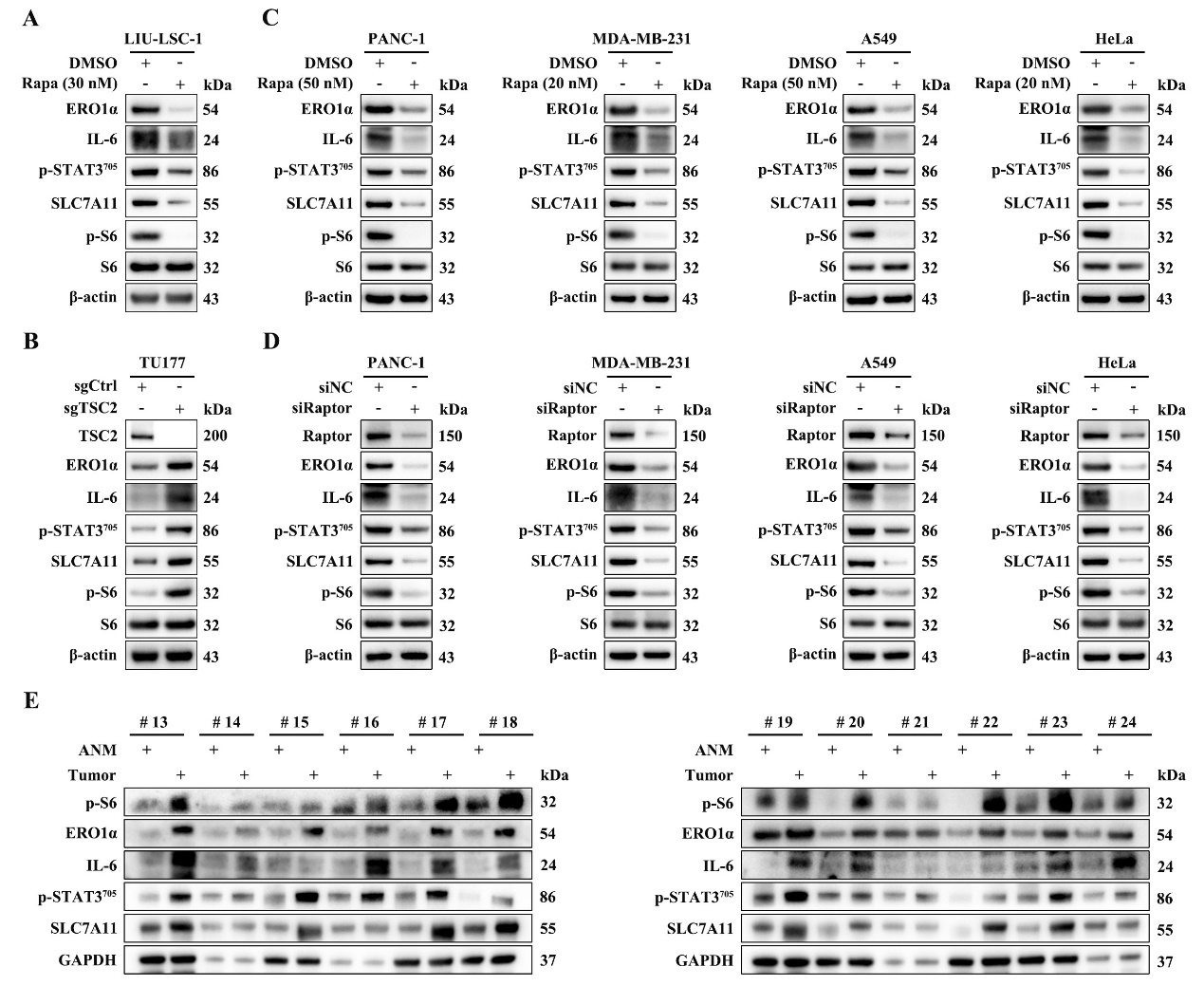
**

**Supplementary Fig. 8: The ERO1α/IL-6/STAT3/SLC7A11 signaling pathway is regulated by mTORC1 signaling in human cancer cells.**

(A) LIU-LSC-1 cells were treated with DMSO or rapamycin (30 nM) for 24 hours. (B) Wild-type (sgCtrl) and TSC2 knockout (sgTSC2) TU177 cells. (C) PANC-1, MDA-MB-231, A549, and HeLa cells were treated with DMSO or the indicated concentration of rapamycin for 24 hours. (D) PANC-1, MDA-MB-231, A549, and HeLa cells were transfected with control siRNAs (siNC) or siRNAs against Raptor (siRaptor) for 48 hours. (E) 12 paired LSCC tissues and ANM tissues (No.13-24). (A-E) Cell lysates were subjected to immunoblotting with the indicated antibodies.

**
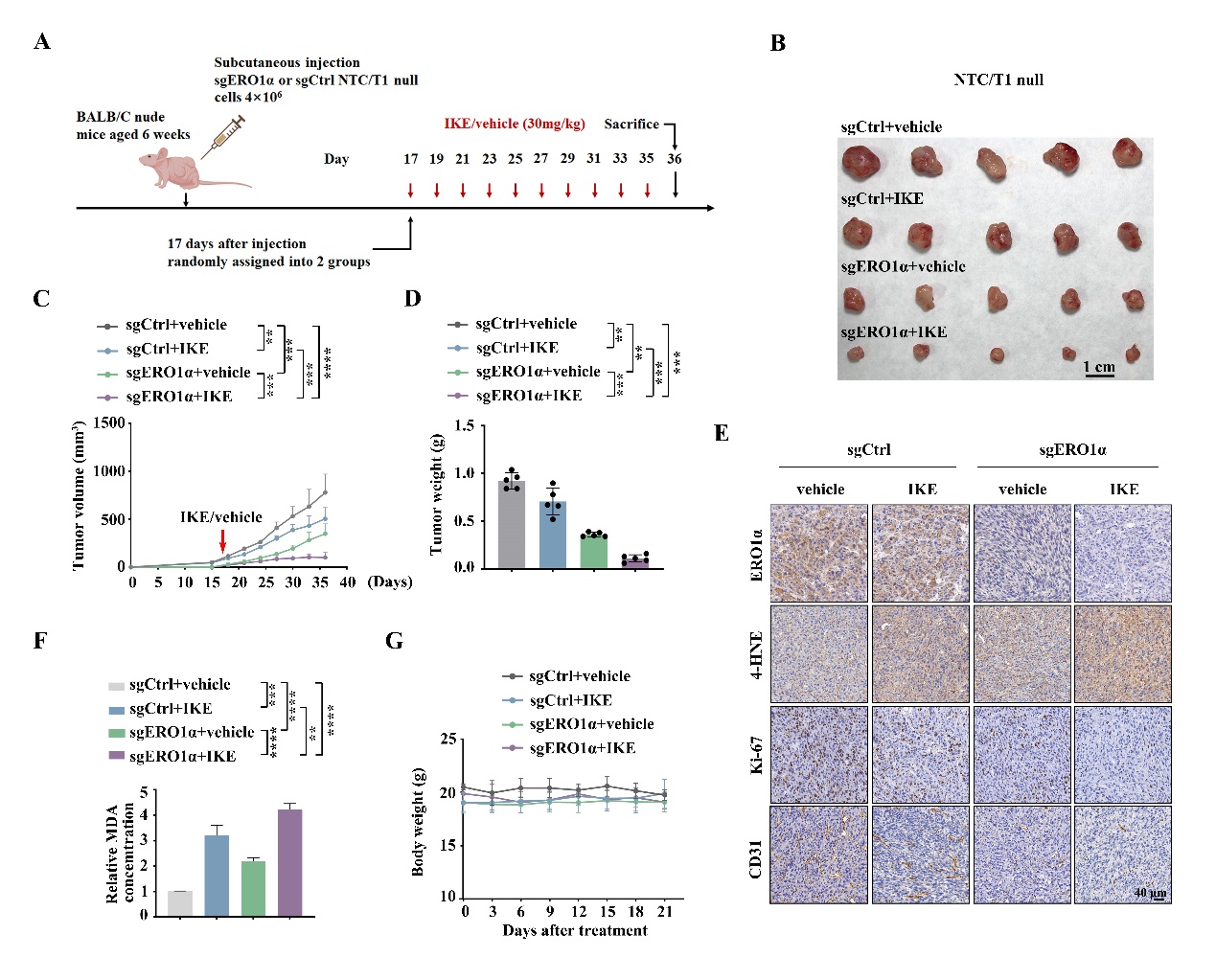
**

**Supplementary Fig. 9: ERO1α depletion sensitizes NTC/T1 null xenograft tumors to IKE.**

(A–G) Xenograft tumors derived from sgERO1α NTC/T1 null cells and the control cells were treated with IKE or vehicle. n = 5 mice per group. (A) Schedule of therapeutic intervention of xenograft tumors using IKE. (B) Tumor images. (C) Tumor growth curve. (D) Tumor weight was measured after tumor excision. (E) IHC staining of ERO1α, 4-HNE, Ki-67, and CD31 in xenograft tumors. Scale bar, 40 μm. (F) The level of MDA in isolated tumors was assayed. (G) Body weight of mice. Error bars indicate mean ± SD (n = 5 mice/group). ***P*<0.01; ****P*<0.001; *****P*<0.0001.


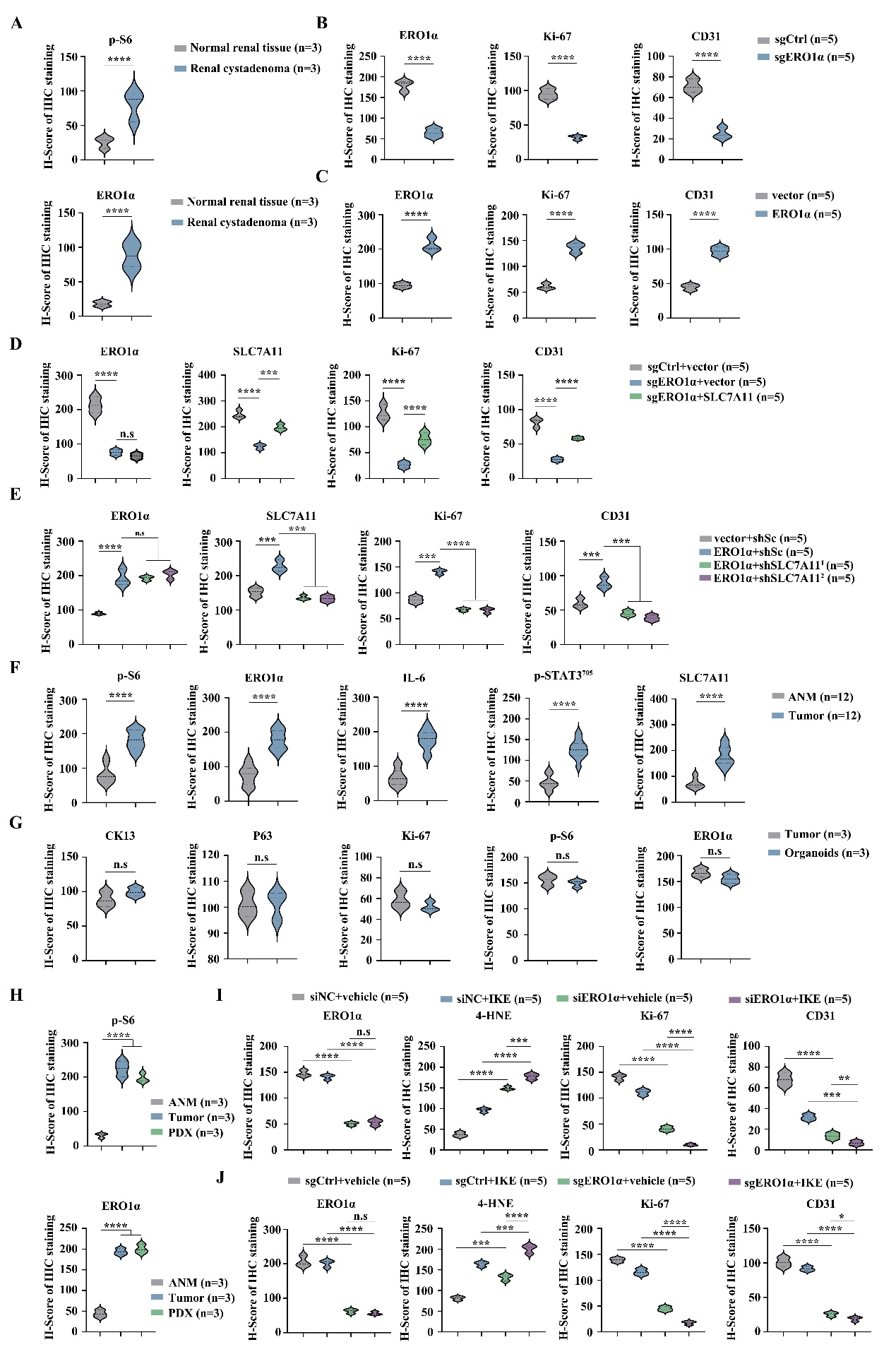


**Supplementary Fig. 10: The quantification of IHC staining.**

The IHC staining results in Fig. 1I, Fig. 2N, Fig. 2R, Fig. 5O, Fig. 5T, Fig. 7D, Fig. 8B, Fig. 8E, Fig. 8J, and Supplementary Fig. 9E were assessed using H-scores, and the quantitative analysis results are presented in A, B, C, D, E, F, G, H, I, and J respectively. Error bars indicate mean ± SD. **P*<0.05; ***P*<0.01; ****P*<0.001; *****P*<0.0001. n.s: no significance.
