# Supplementary material for: Augmented ERO1α upon mTORC1 activation induces ferroptosis resistance and tumor progression via upregulation of SLC7A11
Source: J Exp Clin Cancer Res. 2024 Apr 13;43:112. doi: 10.1186/s13046-024-03039-2 (PMC11015652; doi:10.1186/s13046-024-03039-2)
Supplement: Supplementary file 5 — Supplementary Material 5. [file 13046_2024_3039_MOESM5_ESM.docx]

**Supplementary Materials for Reviewer Only**

**
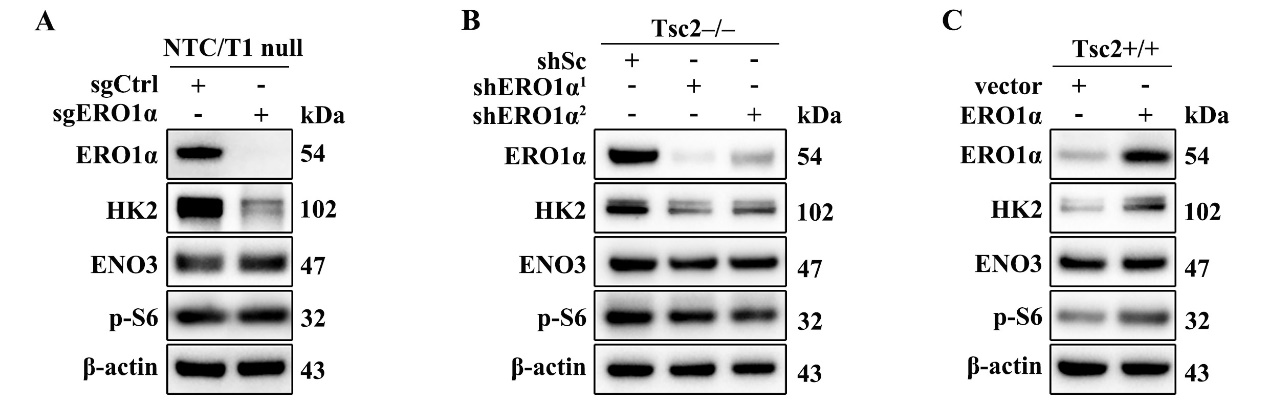
**

**Supplementary Fig. 11: The effect of knockout, knockdown, or overexpression of ERO1α on the expression of HK2, ENO3, and p-S6.**

(A) sgERO1α and sgCtrl NTC/T1 null cells. (B) ERO1α-knockdown (shERO1α^1^ and shERO1α^2^) Tsc2−/− MEFs and the control (shSc) cells. (C) ERO1α-overexpressing Tsc2+/+ MEFs and the control (vector) cells. (A-C) Cell lysates were subjected to immunoblotting with anti-ERO1α, anti-HK2 (Cell Signaling Technology, Cat#2867), anti-ENO3 (Abcam, Cat#ab157474), and anti-p-S6 antibodies.
